# Overlaid Conductive Silver Nanowire Networks on Gas Diffusion Electrodes for High‐Performance Electrochemical CO_2_‐to‐C_2+_ Conversion

**DOI:** 10.1002/advs.75003

**Published:** 2026-03-24

**Authors:** Jonghyeok Park, Sungjoo Kim, Yunkyoung Han, Hyunjoon Song

**Affiliations:** ^1^ Department of Chemistry Korea Advanced Institute of Science and Technology Daejeon Republic of Korea

**Keywords:** current collector, electrochemical CO_2_ conversion, gas diffusion electrode flow cell, silver nanowire networks, tandem catalyst

## Abstract

Designing selective and durable electrodes for the electrochemical CO_2_ reduction reaction (eCO_2_RR) requires both efficient charge transport and favorable catalytic interfaces. Conventional carbon‐based gas diffusion electrodes are unstable due to electrolyte flooding, whereas flood‐resistant polytetrafluoroethylene (PTFE) substrates exhibit low electrical conductivity. Herein, a stacked‐electrode architecture is reported to overcome these limitations, comprising PTFE as a porous substrate, Cu_2_O nanocubes as a catalyst layer, and Ag nanowire (Ag NW) networks as a current collector. The catalytic performance is highly dependent on the stacking sequence, with the PTFE/Cu_2_O/Ag configuration achieving remarkable C_2+_ selectivity and durability while exhibiting electrical resistance comparable to that of carbon paper. In alkaline electrolytes, this configuration exhibits a Faradaic efficiency toward C_2+_ (FE_C2+_) of 79% and stable operation for more than 50 h, significantly outperforming conventional carbon paper‐based electrodes. Mechanistic studies reveal that the Ag NW networks not only ensure the coexistence of Cu(0)/Cu(I) species but also generate CO to drive tandem reactions, promoting C_2+_ production. Furthermore, it reaches an even higher FE_C2+_ of 86% while maintaining long‐term stability under neutral electrolytes. These findings provide a versatile strategy for the rational design of durable and selective electrodes for eCO_2_RR.

## Graphical abstract

This work presents a rational electrode design for electrochemical CO_2_ reduction, employing Ag nanowire networks as both current collectors and tandem catalysts. The Ag nanowires are deposited onto the catalyst layer, forming a stacked PTFE/catalyst/Ag nanowires configuration. This design achieves ≈80% Faradaic efficiency toward C_2+_ products in both alkaline and neutral electrolytes with high durability for 50 h.

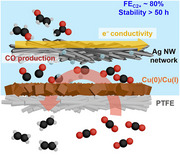

## 1 Introduction

Electrochemical CO_2_ reduction reaction (eCO_2_RR) has emerged as a promising strategy for converting CO_2_ into value‐added chemicals, such as carbon monoxide, ethylene, and ethanol, under ambient conditions [1, 2, 3, 4, 5]. In addition, eCO_2_RR serves as an attractive route for storing renewable energy in the form of chemicals through an environmentally benign process [6, 7, 8, 9]. However, a critical limitation of eCO_2_RR in conventional H‐cell suffers from the inherently low solubility of CO_2_ in the aqueous electrolyte, which restricts its mass transport to the catalyst surface and subsequently lowers the overall production rates of carbon compounds [10, 11, 12, 13].

To overcome this limitation, a gas diffusion electrode (GDE) flow cell has been developed to deliver gaseous CO_2_ directly to the catalyst. It effectively enhances CO_2_ mass transport, thereby significantly improving current densities and bringing the system closer to practical feasibility [14, 15]. Gas diffusion layers (GDLs) require high porosity for gas diffusion with sufficient electrical conductivity for charge transport to establish efficient GDEs. Therefore, carbon‐based GDLs have traditionally been employed due to their scalability and conductivity. However, their tendency to become hydrophilic during eCO_2_RR leads to electrolyte flooding, severely limiting long‐term operation [16, 17, 18, 19, 20]. Recently, fluorinated polymer‐based GDLs such as polytetrafluoroethylene (PTFE) and polyvinylidene fluoride (PVDF) have been explored as a GDL for their intrinsic hydrophobicity, which resists flooding. Yet, its poor electrical conductivity demands the incorporation of conductive components to establish efficient electrical pathways.

Sputtering metals onto PTFE GDLs has been widely adopted to create continuous conductive catalyst layers [21, 22], although this method often lacks catalytic tunability, such as morphology and oxidation state, factors determining the reaction kinetics [23]. In this regard, integrating external current collectors has emerged as a practical approach for improving charge distribution across catalyst layers, particularly for catalysts with inherently low conductivity, such as oxide‐derived copper nanoparticle ensembles. For instance, Burdyny et al. demonstrated that sputtered Cu busbars on PTFE/Cu electrodes ensured uniform current distribution and improved catalyst durability [24]. Similarly, Strasser et al. attached grid‐type current collectors on the catalyst surface, enabling scalable fabrication and enhanced catalytic performance [25]. Yamaguchi et al. coated aluminum onto PVDF GDLs to facilitate current collection for Ag and Cu_2_O catalysts [26].

Nanoparticle‐based catalysts offer several practical advantages, including large surface areas, scalable synthesis, and flexibility to tune their composition and morphology [27]. We note that silver nanowires (Ag NWs) are promising materials for current collectors compatible with diverse catalyst layers. Ag NWs can form continuous, highly conductive networks via simple deposition from colloidal dispersions widely used in transparent conductive electrodes for optoelectronics [28, 29, 30]. Their network structure ensures pore sites available for CO_2_ penetration to catalysts while maintaining electrical pathways, making them particularly suitable for GDL integration. Recently, Raciti et al. employed Ag NW networks on PTFE substrates, achieving Faradaic efficiency (FE) toward CO (FE_CO_) of over 90% under limited conditions in neutral media, demonstrating the potential of an Ag NW‐based configuration for eCO_2_RR [31].

In this study, we present a rational design of stacked electrode architecture comprising PTFE substrates, Ag NWs or conductive carbon as the current collector, and discrete Cu_2_O nanocubes (Cu_2_O NCs) as the catalyst layer. These components were sequentially assembled via spray coating from stable colloidal dispersions, enabling facile fabrication. Notably, the catalytic properties of the electrodes were highly dependent on the stacking sequence of the constituent layers. Among the configurations, the PTFE/Cu_2_O/Ag configuration, with the stacking order of PTFE GDL, Cu_2_O NCs, and Ag NW networks sequentially, exhibited significant C_2+_ selectivity and stability in both alkaline and neutral electrolytes with low overpotential. Operando X‐ray absorption spectroscopy and CO_2_/CO co‐reduction experiments revealed that Cu(0)/Cu(I) mixed oxidation states of the catalyst and in situ generated CO at the Ag NW networks facilitated C─C coupling [32, 33, 34]. These findings highlight the multifunctional role of Ag NW networks, serving as both an efficient current collector and a CO intermediate generator. This multifunctionality enables the development of high‐performance electrode architectures for eCO_2_RR systems.

## 2 Results and Discussion

### 2.1 Constructing Multi‐Layered Gas Diffusion Electrodes

**FIGURE 1:**
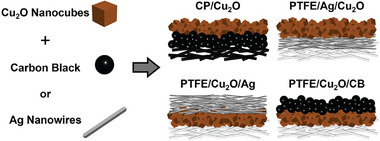
Fabrication of GDEs with four distinct configurations. (Left) Building components for multi‐layered GDEs. (Right) Four types of electrode configurations with diverse stacking layers: CP/Cu_2_O, PTFE/Ag/Cu_2_O, PTFE/Cu_2_O/Ag, and PTFE/Cu_2_O/CB.

For fabricating GDEs incorporating catalyst nanoparticles, we designed electrode architectures consisting of three essential components. A PTFE substrate was employed as the hydrophobic porous GDL, and Cu_2_O NCs as the active catalyst layer. While carbon black (CB) is conventionally employed as a conductive material, we introduced Ag NW networks as an alternative current collector (Figure 1, left). As a standard electrode, Cu_2_O NCs were deposited on carbon paper (denoted as CP/Cu_2_O). In addition, three distinct stacking configurations were fabricated on the PTFE substrate (Figure 1, right). First, Ag NWs were deposited prior to Cu_2_O NCs (PTFE/Ag/Cu_2_O), thereby exposing the catalyst directly to the electrolyte. The Ag NW layer on PTFE (PTFE/Ag) was intended to mimic the carbon paper. The second followed the reverse order, depositing Cu_2_O NCs first and then overlaying Ag NW networks (PTFE/Cu_2_O/Ag), with the conductive layer positioned on top of the catalyst. For comparison, a third configuration was prepared using a CB layer onto Cu_2_O NCs on PTFE (PTFE/Cu_2_O/CB).

### 2.2 Synthesis and Characterization of Catalyst Electrodes

**FIGURE 2:**
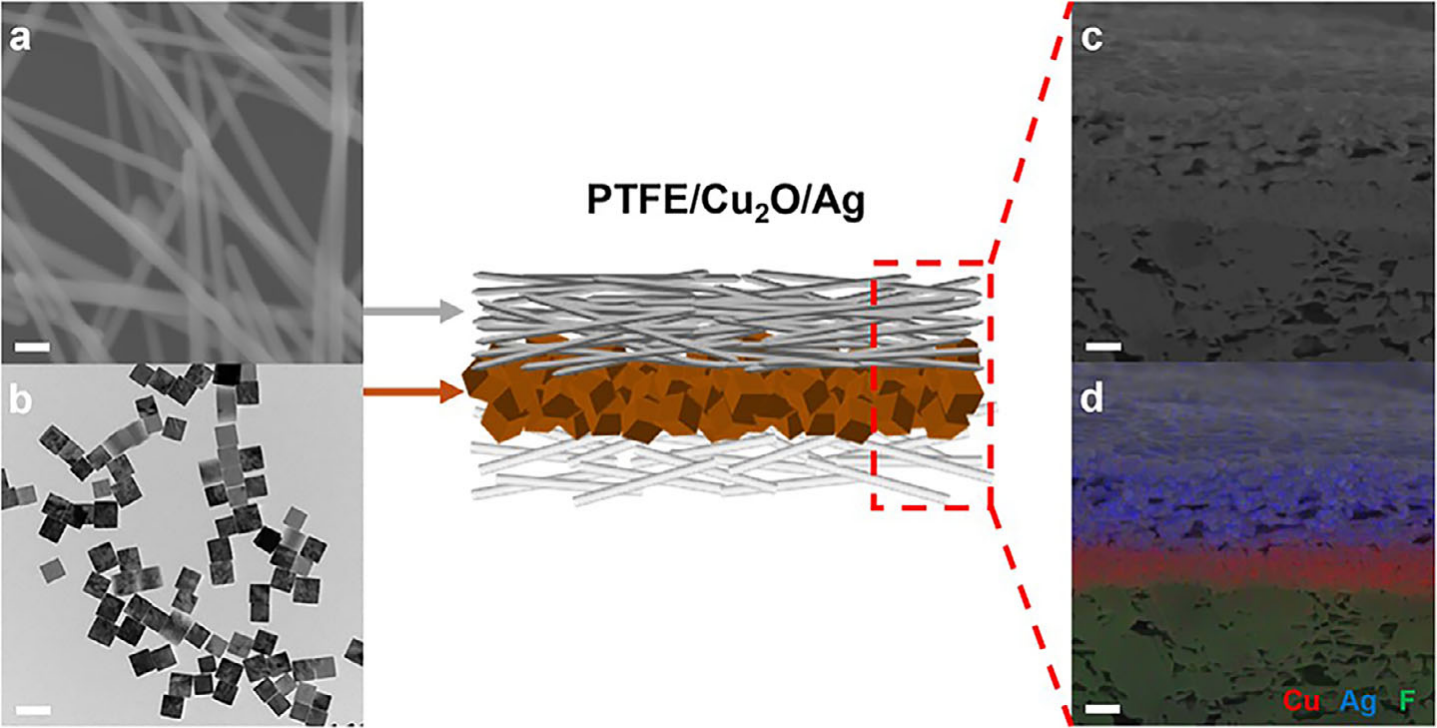
Characterization of PTFE/Cu_2_O/Ag electrode and its components. (a) An SEM image of Ag NWs. (b) A TEM image of Cu_2_O NCs. (c) Cross‐sectional SEM image and (d) corresponding EDS elemental mapping of PTFE/Cu_2_O/Ag. The scale bars represent (a, b) 100 nm and (c, d) 200 nm.

Ag NWs and Cu_2_O NCs were synthesized via a modified polyol process in the presence of poly(vinylpyrrolidone) (PVP) [31, 35]. Detailed synthetic methods were described in Supporting Information. Scanning electron microscopy (SEM) image confirmed the formation of straight Ag NWs (Figure 2a) with an average thickness of 38 ± 6 nm and a length of 3.0 ± 0.6 µm (Figure S1a, b). Transmission electron microscopy (TEM) revealed highly uniform Cu_2_O NCs with an average edge size of 81 ± 5 nm (Figure 2b; Figure S1c). X‐ray diffraction (XRD) verified the crystal phases of metallic Ag (PDF #01‐077‐6577) and copper(I) oxide (PDF #01‐078‐2076), respectively (Figure S2). Then, we characterized the PTFE/Cu_2_O/Ag electrode. Cross‐sectional SEM imaging of the fabricated electrodes clearly displayed distinct multilayer stacks, with each layer deposited uniformly and with consistent thickness. For the PTFE/Cu_2_O/Ag configuration, the average thicknesses of the Cu_2_O NCs and Ag NW layers were measured to be 240 and 500 nm, respectively (Figure 2c). Energy‐dispersive spectroscopy (EDS) elemental mapping further distinguished the layers by elemental signatures: PTFE (green, F), Cu_2_O NCs (red, Cu), and Ag NW networks (blue, Ag) (Figure 2d). Ag NW deposition yielded interconnected networks with uniform density, as evidenced by the top‐view SEM image (Figure S3).

### 2.3 eCO_2_RR in Alkaline Electrolytes

The eCO_2_RR process was performed in a GDE flow cell (Figure S4) using the catalyst electrodes in a 1.0 м KOH aqueous electrolyte. Chronopotentiometry was employed to drive the reaction at applied current densities (*j*_total_) ranging from −400 to −100 mA cm^−2^ (see Supporting Information for details). All reaction products were quantitatively analyzed using gas chromatography (GC) and high‐performance liquid chromatography (HPLC).

**FIGURE 3:**
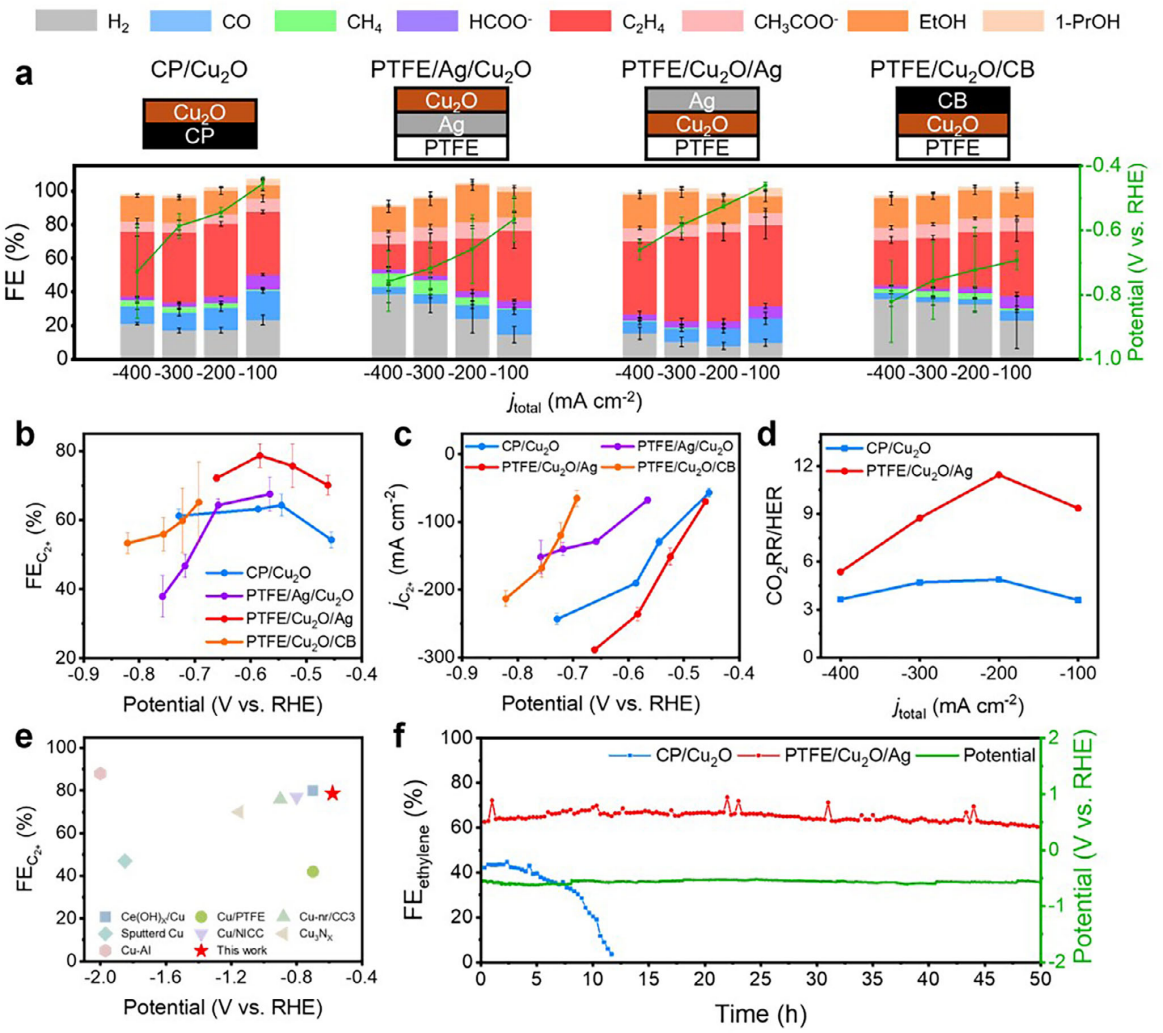
Reaction study under alkaline media. (a) FE and applied potential versus total current density, and (b) FE_C2+_ and (c) C_2+_ partial current density versus applied potential for four electrode configurations in 1.0 м KOH. (d) The ratio of CO_2_RR/HER versus total current density for CP/Cu_2_O and PTFE/Cu_2_O/Ag. (e) Comparison of FE_C2+_ versus applied potential of PTFE/Cu_2_O/Ag and the catalysts reported in literature (Table S6) in alkaline media. (f) FE_ethylene_ and potential change over 50 h using CP/Cu_2_O and PTFE/Cu_2_O/Ag in 1.0 м KOH.

The eCO_2_RR products were generated simultaneously, including hydrogen, C_1_ (CO, CH_4_, and formate), and C_2+_ (ethylene, acetate, ethanol, and 1‐propanol) products at the applied potentials. Among them, ethylene was the predominant product under optimized conditions across all electrode configurations (Figure 3a). Comprehensive electrochemical performance data are summarized in Tables S1–S4. As a conventional model electrode, CP/Cu_2_O achieved a maximum FE of 43% toward ethylene (FE_ethylene_) and 64% toward C_2+_ products (FE_C2+_) at −200 mA cm^−2^. In contrast, PTFE/Ag/Cu_2_O displayed reduced ethylene and C_2+_ selectivity and higher hydrogen evolution, particularly at elevated current densities. On the other hand, the inverse stacking configuration, PTFE/Cu_2_O/Ag, markedly enhanced the selectivity for ethylene and C_2+_ products, effectively suppressing hydrogen generation across the entire current‐density range. This electrode achieved the maximum FE_ethylene_ and FE_C2+_ values of 53% at −200 mA cm^−2^ and 79% at −300 mA cm^−2^, respectively, while minimizing the FE toward hydrogen (FE_H2_) to only 8% at −200 mA cm^−2^. Conversely, PTFE/Cu_2_O/CB exhibited inferior performance of C_2+_ products with a maximum FE_C2+_ value of 69% at −100 mA cm^−2^.

To confirm the tendency of FE_C2+_ depending on potential, FE_C2+_ was plotted as a function of applied potential for each electrode (Figure 3b). The PTFE/Cu_2_O/Ag maintained a high FE_C2+_ across the potential range compared to the other electrode configuration, reaching a maximum of 79% at −0.58 V versus reversible hydrogen electrode (RHE). The potential range associated with the applied current density was comparable to that of CP/Cu_2_O, suggesting similar electrode resistance. In contrast, PTFE/Ag/Cu_2_O and PTFE/Cu_2_O/CB exhibited higher potentials with reduced selectivity. To evaluate the electrical conductivity of the current‐collecting layers, sheet resistance was measured. CP/Cu_2_O, PTFE/Ag/Cu_2_O, and PTFE/Cu_2_O/Ag exhibited low sheet resistances of 1.1, 0.31, and 0.64 Ω/sq, respectively, while PTFE/Cu_2_O/CB displayed a dramatically higher resistance of 2860 Ω/sq (Figure S5). These results confirmed that Ag NW networks formed continuous conductive pathways for electron transport, with the potential to replace carbon paper by overcoming charge transport limitations. To elucidate the charge transport dynamics and evaluate the mass transport properties of the PTFE‐based architecture, in situ potentiostatic electrochemical impedance spectroscopy (PEIS) was performed at −0.6 V versus RHE. The equivalent circuit models utilized for the different electrode configurations are detailed in Figure S6. The modeling revealed that PTFE/Cu_2_O/Ag exhibited a minimized Ohmic resistance (*R*_s_ = 2.92 Ω) and a significantly reduced low‐frequency charge transfer resistance (*R*_ct2_ = 0.256 Ω) compared to the other configurations. These results indicate that the overlaid Ag NW network facilitates superior electron transport and promotes efficient CO_2_ mass transfer to the active sites (Figure S7 and Table S5). Importantly, PTFE/Cu_2_O/Ag achieved high conductivity and superior C_2+_ selectivity, resulting in the highest partial current density for C_2+_ products (*j*_C2+_) among all electrode configurations. The maximum *j*_C2+_ reached −290 mA cm^−2^ at −0.66 V versus RHE (Figure 3c; Figure S8), whereas PTFE/Ag/Cu_2_O and PTFE/Cu_2_O/CB delivered lower values, consistent with their less effective current collection behaviors.

Because eCO_2_RR and hydrogen evolution reaction (HER) are competitive on active catalyst surfaces, the selectivity ratio (CO_2_RR/HER) was evaluated (Figure 3d). At −200 mA cm^−2^, PTFE/Cu_2_O/Ag achieved a ratio of 11.5, 2.3 times higher than that of CP/Cu_2_O. This result highlights that PTFE/Cu_2_O/Ag not only promotes CO_2_RR but also suppresses HER more effectively in the same total current density.

Overall, under alkaline conditions in a flow cell system, PTFE/Cu_2_O/Ag stands out as one of the most efficient catalysts for C_2+_ production, combining high FE_C2+_ with low overpotential (Figure 3e and Table S6).

Long‐term stability tests emphasized the robustness of this configuration (Figure 3f). In 1.0 м KOH at −200 mA cm^−2^, the FE_ethylene_ of PTFE/Cu_2_O/Ag remained ≈60% after 50 h (red line), whereas CP/Cu_2_O rapidly deactivated within 12 h (blue line). The enhanced durability of PTFE/Cu_2_O/Ag is attributed to the protective role of the overlaid Ag NW networks, which shield Cu_2_O NCs from degradation while simultaneously providing efficient electron transport [36]. This strategy is consistent with other protective overlayers, such as carbon shells or SiC‐Nafion coatings, that have been reported to improve catalyst lifetimes [4, 37]. Hence, the Ag NW networks serve multiple functions as a robust protective overlayer that stabilizes the catalysts and a conductive pathway that minimizes resistance. To elucidate the structural and chemical evolution of the Cu_2_O NCs and Ag NWs, ex situ TEM, SEM and XRD analyses were performed following the 50 h stability test. The observations revealed that the Cu_2_O NCs largely maintained their primary cubic morphology, although local surface reconstruction was observed at the corners and edges by the reduction of Cu_2_O to Cu(0) (Figure S9a). High resolution TEM (HRTEM) imaging and XRD analysis corroborated the coexistence of metallic Cu and Cu_2_O phases within the catalyst layer (Figures S9b and S10). In contrast, the Ag NW network demonstrated exceptional structural integrity, retaining its original morphology without evidence of sintering or fragmentation (Figure S9c–e). Corresponding ex situ XRD patterns exhibited intense peaks characteristic of metallic Ag, with no detectable oxide phases, even after prolonged exposure to the electrolyte (Figure S10). These findings confirm that both the Cu active sites and the overlaid Ag NW scaffold possess high electrochemical robustness under alkaline conditions, facilitating the durable electrocatalytic performance observed over 50 h of operation.

In alkaline media, the accumulation of (bi)carbonate salts typically indices electrode flooding by occluding gas transport channels, thereby limiting long‐term catalytic stability. While significant crystalline phases of (bi)carbonate were identified in the XRD spectrum following the 50 h stability test (Figure S10), the SEM image revealed that these precipitates were predominantly localized on the Ag NW network (Figure S9c). Consequently, the porous architecture remained largely unblocked, ensuring that gas permeability was maintained throughout the operational window.

### 2.4 Observing Oxidation States of Cu Catalysts via Operando X‐ray Absorption Near‐Edge Structure

**FIGURE 4:**
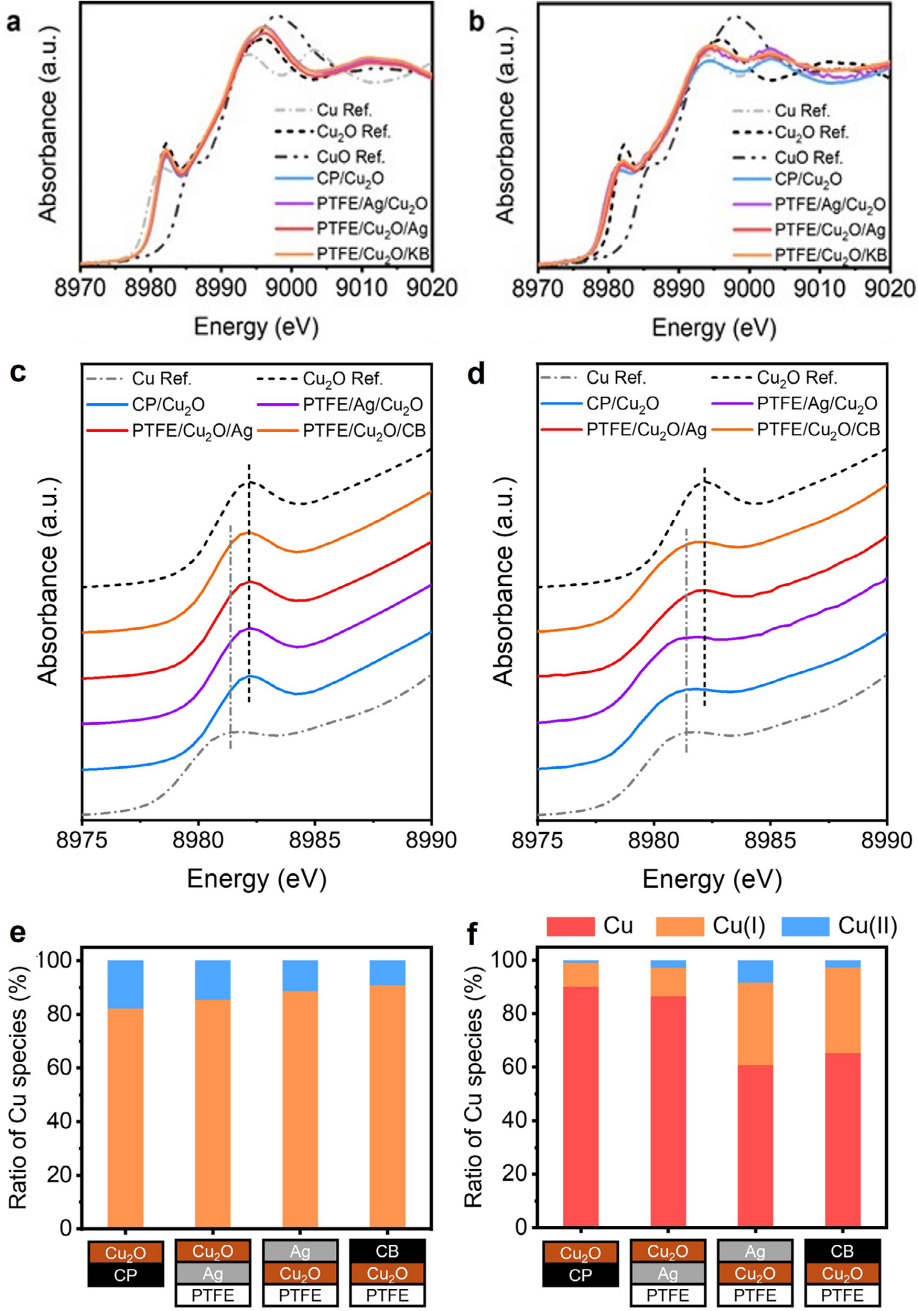
Estimation of Cu oxidation states before and during eCO_2_RR. Operando Cu K‐edge XANES of the catalyst electrodes (a) as prepared and (b) during eCO_2_RR. Enlarged shoulder peaks of Cu K‐edge XANES at (c) as prepared and (d) during eCO_2_RR. The ratio of Cu species for the electrodes (e) as prepared and (f) during eCO_2_RR via LCF.

Understanding the nature of the active catalyst species is essential for establishing structure‐reactivity relationships under working electrochemical conditions. We performed operando X‐ray absorption near‐edge structure (XANES) experiments at the Cu K‐edge region in a 1.0 м KOH electrolyte. For all as‐prepared samples, the shapes of XANES data matched those of Cu_2_O reference, indicating that Cu_2_O NCs were maintained in the initial state (Figure 4a). When the potential was applied, Cu_2_O NCs were reduced to Cu(0), with the degree of reduction varying among the electrode configurations. XANES curves of CP/Cu_2_O and PTFE/Ag/Cu_2_O followed that of Cu(0) reference, while PTFE/Cu_2_O/Ag and PTFE/Cu_2_O/CB partly retained the characteristic features of Cu_2_O, evidenced by suppressed absorbance near 9005 eV (Figure 4b). To observe the detailed differences in the reduction, the shoulder peak ranges were expanded (Figure 4c, d). The dominant Cu species was Cu(I), exhibiting a characteristic absorption shoulder at 8982 eV, identical to that of the pristine Cu_2_O reference (black dashed line). After applying the potential, the absorption shoulders shifted toward lower energies, approaching the Cu(0) reference at 8981 eV (gray dashed‐dot line). Both CP/Cu_2_O and PTFE/Ag/Cu_2_O exhibited extensive reduction to metallic Cu. In contrast, PTFE/Cu_2_O/Ag and PTFE/Cu_2_O/CB displayed broader features primarily centered on Cu_2_O, indicating that some Cu(I) species remained unaltered. For accurate quantification of Cu species, linear combination fitting (LCF) analysis (Figure S11 and Table S7) was performed, indicating that Cu(I) was the predominant species on all electrodes before reaction (Figure 4e). The catalysts on CP/Cu_2_O and PTFE/Ag/Cu_2_O were reduced almost entirely to metallic Cu, leaving 10% residual Cu(I) during eCO_2_RR. By contrast, 30% of Cu(I) species persisted in PTFE/Cu_2_O/Ag and PTFE/Cu_2_O/CB (Figure 4f). These results suggest that the overlayers—either the Ag NW networks or CB—partially shield the complete reduction of the catalyst surface, thereby stabilizing Cu(I) during operation [38]. The coexistence of Cu(0) and Cu(I) has been widely recognized as beneficial for C_2+_ formation, as mixed‐valence surfaces provide adsorption sites that facilitate C─C coupling [39, 40]. Wu et al., for instance, reported that CO_atop_ and CO_bridge_ species coexisted on a mixed Cu(0)/Cu(I) interface, promoting the dimerization pathway toward multicarbon products [41]. Meanwhile, the operando Ag K‐edge XANES confirmed that the oxidation state of Ag NWs remained unchanged, maintaining Ag(0) throughout eCO_2_RR (Figure S12).

### 2.5 Electrochemical Reduction of the CO_2_/CO Mixture

The oxidation state of the Cu_2_O NCs during reaction was similar between PTFE/Cu_2_O/Ag and PTFE/Cu_2_O/CB, suggesting that the stacking order of the electrode configuration governs the catalytic conditions. Despite this similarity, PTFE/Cu_2_O/Ag exhibited markedly superior performance in terms of FE_C2+_, overpotential, and *j*_C2+_, highlighting the decisive role of the overlaid Ag NW networks in eCO_2_RR. Before elucidating the role of the Ag NW network, the eCO_2_RR of the Ag NW network was demonstrated. When Ag NWs were deposited solely on PTFE (PTFE/Ag), the electrode exclusively produced CO, with a FE_CO_ exceeding 90% within the same potential range as measured in PTFE/Cu_2_O/Ag, which was actively involved in the reaction (Figure S13). This observation implies that in situ generated CO on the Ag NW overlayer significantly contributes to the enhanced performance of the Cu_2_O catalyst [42].

**FIGURE 5:**
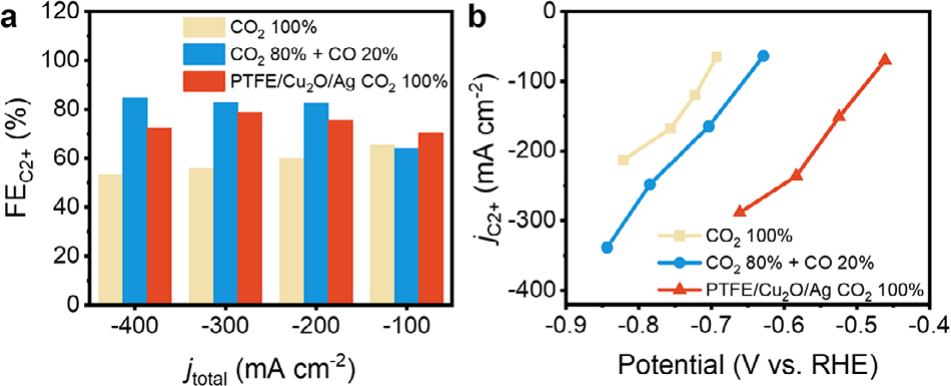
Exploring the tandem effect of Ag NW networks. (a) FE_C2+_ versus the total current density and (b) C_2+_ partial current density versus applied potential with pure CO_2_ and a mixture of 80% CO_2_ and 20% CO using PTFE/Cu_2_O/CB, compared to pure CO_2_ using PTFE/Cu_2_O/Ag.

To validate the significance of insitu generated CO, co‐reduction experiments of CO_2_ and CO were conducted using PTFE/Cu_2_O/CB, where the top CB layer serves as a current collector but does not produce CO (Figure 5a). When pure CO_2_ was fed to PTFE/Cu_2_O/CB, FE_C2+_ ranged from 50%–65%, with a maximum of 65% at −100 mA cm^−2^ (beige columns). With the feeding of a mixed gas consisting of 80% CO_2_ and 20% CO, FE_C2+_ increased significantly to 65%–85%, especially at high current densities, reaching a maximum of 85% at −400 mA cm^−2^ (blue columns). These FEs closely matched those obtained with PTFE/Cu_2_O/Ag under 100% CO_2_ feeding (red columns), indicating that the overlaid Ag NW network provide a local environment comparable to that of the underlying Cu_2_O catalyst under CO co‐feeding. Importantly, the applied potential range for PTFE/Cu_2_O/CB was not significantly changed despite CO addition, confirming that the increase in C_2+_ partial current density arose from the tandem CO supply (Figure 5b). In contrast, PTFE/Cu_2_O/Ag exhibited higher *j*_C2+_ at lower overpotentials. It indicates that the Ag NW networks effectively collect current and produce CO during the reaction, owing to their low sheet resistance.

These findings are consistent with the previous reports that incorporating CO‐producing catalysts, such as Ag and cobalt phthalocyanine, into overlayer configurations enhances C_2+_ selectivity in eCO_2_RR [43, 44, 45]. Furthermore, previous reports on both simulations and experiments have demonstrated that placing additive CO‐producing layers above Cu catalysts facilitates the effective back diffusion of CO, thereby maximizing its utilization [46, 47]. Taken together, Ag NW networks play a synergistic role, acting as active CO‐generating catalysts, thereby creating a relatively CO‐sufficient environment for selective C_2+_ formation in the PTFE/Cu_2_O/Ag configuration.

### 2.6 eCO_2_RR in Neutral Electrolytes

PTFE/Cu_2_O/Ag demonstrated superior selectivity and activity compared to other electrode configurations in alkaline media. While high pH suppresses HER and favors CO dimerization to C_2+_ products, the abundance of hydroxide ions also promotes (bi)carbonate formation, leading to severe salt precipitation and CO_2_ depletion. To mitigate these drawbacks, neutral electrolytes have been proposed as an alternative; however, FE_C2+_ is typically lower than in alkaline environments [48]. To assess the versatility of our configuration across different pH conditions, PTFE/Cu_2_O/Ag was tested in a neutral electrolyte and compared to CP/Cu_2_O.

**FIGURE 6:**
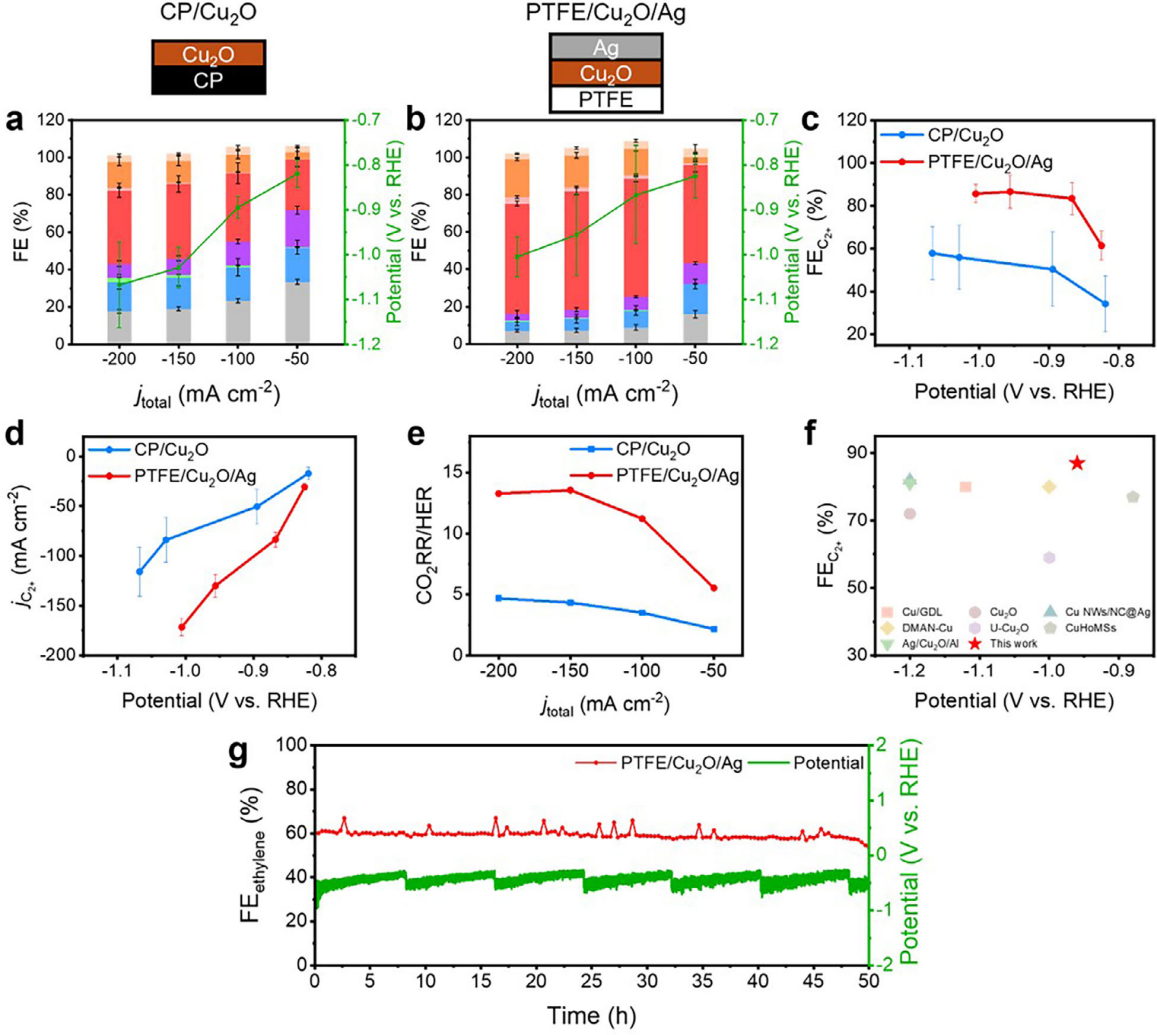
Reaction study under neutral media. FEs and applied potentials versus total current densities for (a) CP/Cu_2_O and (b) PTFE/Cu_2_O/Ag in 1.0 м KHCO_3_. The legend for each product is identical to that in Figure 3. The plots of (c) FE_C2+_ versus potential, (d) C_2+_ partial current density versus potential, and (e) the ratio of CO_2_RR/HER for CP/Cu_2_O and PTFE/Cu_2_O/Ag, respectively. (f) Comparison of FE_C2+_ versus applied potential of PTFE/Cu_2_O/Ag and the catalysts reported in literature (Table S10) in neutral media. (g) FE_ethylene_ and potential change over 50 h using PTFE/Cu_2_O/Ag in 1.0 м KHCO_3_.

eCO_2_RR was carried out in 1.0 м KHCO_3_ at current densities ranging from −200 to −50 mA cm^−2^. CP/Cu_2_O delivered FE_ethylene_ and FE_C2+_ of 40% and 58%, respectively, at −200 mA cm^−2^, which is slightly lower yet comparable to its performance in alkaline media (Figure 6a and Table S8). By contrast, PTFE/Cu_2_O/Ag achieved FE_ethylene_ and FE_C2+_ of 59 and 86%, respectively, at the same current density (Figure 6b and Table S9), surpassing CP/Cu_2_O and even outperforming all configurations tested under alkaline conditions. HER was also strongly suppressed, with FE_H2_ values of only 7%–10% compared to > 15% for CP/Cu_2_O.

Regarding potential dependence, PTFE/Cu_2_O/Ag maintained FE_C2+_ of > 80% over a wide potential window of −0.87 to −1.02 V versus RHE, whereas CP/Cu_2_O remained below 60% throughout (Figure 6c). *j*_C2+_ were more than double those of CP/Cu_2_O at identical potentials (Figure 6d; Figure S14). The CO_2_RR/HER ratio reached 13.6 for PTFE/Cu_2_O/Ag at −150 mA cm^−2^, over three times higher than 4.4 for CP/Cu_2_O, indicating the effects of CO_2_RR promotion and HER suppression that were proved in alkaline media (Figure 6e). PTFE/Ag generated CO with the FE_CO_ of ≈100%, particularly at high potentials, indicative of similar CO production from the Ag NW networks and the tandem reaction contributing to C_2+_ formation under neutral conditions (Figure S15).

These results are remarkable given that C_2+_ selectivity is generally considered poor in neutral electrolytes due to the low hydroxide concentration [49, 50]. We attribute the exceptional performance of PTFE/Cu_2_O/Ag to the Ag NW overlayer, which not only serves as an efficient current collector but also produces CO in situ, thereby enabling tandem reaction. These effects enhance C_2+_ selectivity even under neutral conditions. Compared with catalysts reported in the literature, our electrode architecture exhibits the highest FE_C2+_ and one of the lowest overpotentials under neutral electrolyte conditions (Figure 6f and Table S10).

Long‐term stability of PTFE/Cu_2_O/Ag was tested under 1.0 м KHCO_3_ by applying chronopotentiometry at −150 mA cm^−2^ (Figure 6g). FE_ethylene_ was maintained at ∼60% over 50 h, indicating that this electrode configuration is consistently robust and insensitive to pH conditions in both neutral and alkaline electrolytes. Following the 50 h stability test in 1.0 м KHCO_3_, TEM and HRTEM imaging of the Cu_2_O NCs within the catalyst layer confirmed the retention of their cubic morphology, although some minor edge rounding was observed, consistent with the morphological evolution under the alkaline condition (Figure S16a, b). The structural robustness and chemical stability of the Ag NW network were also validated via electron microscopy, which showed no evidence of structural degradation or detachment (Figure S16c–e). These microscopic observations were further corroborated by the XRD spectrum (Figure S17), which showed no significant change in peak positions or patterns. Notably, the characteristic peaks for carbonate species were absent in the spectrum, indicating that salt‐induced surface occlusion is negligible in 1.0 м KHCO_3_ even after prolonged operation. This emphasizes the superior operational stability of the integrated architecture in near‐neutral environments.

## 3 Conclusions

To address the demand for efficient current collectors in PTFE‐based GDEs, we employed Ag NW networks, which provide a continuous electron‐conducting pathway and intrinsic porosity. We developed a stacked electrode configuration of PTFE/Cu_2_O/Ag, in which Cu_2_O NC and Ag NW layers were sequentially deposited on the PTFE GDLs. Under alkaline conditions, this electrode delivered a FE_C2+_ of 79% with operational stability exceeding 50 h, representing a 1.5‐fold enhancement in selectivity and a 10‐fold improvement in durability compared with conventional carbon paper electrodes. Notably, in neutral electrolytes, the electrode achieved an even higher FE_C2+_ of 86% while maintaining long‐term stability, emphasizing its versatility across a wide pH range.

Operando X‐ray absorption spectroscopy confirmed the coexistence of Cu(0) and Cu(I) in PTFE/Cu_2_O/Ag, stabilized by overlaid Ag NW networks, which are catalytically active during the reaction. Furthermore, CO_2_/CO co‐reduction experiments demonstrated that in situ‐generated CO from the Ag NW networks facilitated the tandem reaction, thereby enhancing C_2+_ product formation and reducing overpotential due to the low electrical resistance of the Ag NW networks. These findings establish the Ag NW networks serving as efficient current collectors and tandem catalysts.

Overall, this work introduces a universal electrode architecture particularly suited for nanoparticle‐based catalyst systems. The electrode design, with catalyst layers sandwiched by PTFE and Ag NW networks, provides a pathway toward durable and selective eCO_2_RR, offering practical applications for energy conversion technologies.

## Author Contributions

J.P. and H.S. designed all the experiments. J.P. conducted the experiments and performed the analysis. S.K. obtained TEM images and XRD data. J.P. and Y.H. conducted the operando XANES experiment. J.P. and H.S. wrote the manuscript.

## Conflicts of Interest

The authors declare no conflict of interest.

## Supporting information




**Supporting File**: advs75003‐sup‐0001‐SuppMat.docx.

## Data Availability

The data that support the findings of this study are available from the corresponding author upon reasonable request.
