# Supplementary material for: Overlaid Conductive Silver Nanowire Networks on Gas Diffusion Electrodes for High‐Performance Electrochemical CO2‐to‐C2+ Conversion
Source: Adv Sci (Weinh). 2026 Mar 24;13(32):e75003. doi: 10.1002/advs.75003 (PMC13252605; doi:10.1002/advs.75003)
Supplement: Supplementary file 1 — Supporting File: advs75003‐sup‐0001‐SuppMat.docx. [file ADVS-13-e75003-s001.docx]

Supporting Information

**Overlaid Conductive Silver Nanowire Networks on Gas Diffusion Electrodes for High-Performance Electrochemical CO_2_-to-C_2+_ Conversion**

*Jonghyeok Park, Sungjoo Kim, Yunkyoung Han, and Hyunjoon Song**

Department of Chemistry, Korea Advanced Institute of Science and Technology, Daejeon 34141, Republic of Korea

E-mail: [hsong@kaist.ac.kr](mailto:hsong@kaist.ac.kr)

**Experimental Section**

*Chemicals and Materials* Silver nitrate (AgNO_3_), poly(vinylpyrrolidone) (PVP, M_w_ = ~55,000), ethylene glycol (C_2_H_4_(OH)_2_, EG), iron(III) nitrate nonahydrate (Fe(NO_3_)_3_·9H_2_O), sodium chloride (NaCl), copper acetylacetonate (Cu(acac)_2_), 1,5-pentanediol (HO(CH_2_)_5_OH), and Nafion® perfluorinated resin solution (5 wt%) were purchased from Sigma Aldrich. 2-propanol ((CH_3_)_2_CHOH) was acquired from Junsei. Potassium hydroxide (KOH, 93%), ethanol, and acetone were obtained from Daejung. Platinum gauze was sourced from Thermo Scientific. Ketjen Black powder was purchased from Lion. Carbon paper (Sigracet 39BB) was purchased from Fuel Cell Store. Polytetrafluoroethylene (PTFE) membrane was obtained from Sterlitech. All chemicals were used without further purification.

*Synthesis of Ag Nanowires (Ag NWs)* Ag NW synthesis was according to the literature^[1]^. EG (40 mL) was added to a 100 mL double-neck round-bottom flask. The flask was heated to 180 °C with vigorous stirring in an oil bath. Four solutions were prepared for sequential injection. **Solution 1** was prepared by dissolving NaCl (0.26 g) in EG (20 mL). **Solution 2** consisted of Fe(NO_3_)_3_·9H_2_O (0.14 g) dissolved in EG (20 mL). **Solution 3** encompassed PVP (1.1 g) dissolved in EG (20 mL), while **Solution 4** comprised AgNO_3_ (1.1 g) dissolved in EG (20 mL). Sequential injections were conducted precisely as follows: 0.050 mL of **Solution 1**, 0.025 mL of **Solution 2**, 5.2 mL of **Solution 3**, and 5.2 mL of **Solution 4**. The reaction mixture was stirred for 15 min at 180 °C. After cooling the reaction mixture to room temperature, the product was washed with ethanol and acetone three times with the aid of centrifugation at 10,000 rpm for 30 min. The final product was stored in ethanol.

*Synthesis of Cu_2_O Nanocubes (Cu_2_O NCs)* Cu_2_O NC synthesis was according to the literature^[2]^. PVP (5.3 g) was dissolved in 1,5-pentanediol (45 mL) in a 100 mL double-necked round-bottom flask. Cu(acac)_2_ (1.1 g) was dissolved in 1,5-pentanediol (15 mL) in a 30 mL vial. The flask temperature was raised to 220 °C under an inert atmosphere. The Cu(acac)_2_ solution was injected into the flask using a gastight syringe. The reaction mixture was stirred for 14 min at 220 °C. After cooling the reaction mixture to room temperature, the product was washed with ethanol and acetone three times with the aid of centrifugation at 10,000 rpm for 40 min. The final product was stored in ethanol.

*Characterization* Transmission electron microscopy (TEM) images were obtained using an FEI Tecnai G2 F30 S-Twin. Scanning electron microscopy (SEM) images and energy-dispersive X-ray spectroscopy (EDS) elemental mapping analysis were conducted on a Hitachi SU5000 and JSM-IT800, respectively. X-ray diffraction (XRD) was measured with a Rigaku SmartLab and Ultima IV. Inductively coupled plasma-optical emission spectroscopy (ICP-OES, Agilent) was applied to analyze the density of catalyst samples in the product solution. Sheet resistance was measured with CMT-SR1000N.

*Fabrication of Gas Diffusion Electrodes (GDEs)* The GDEs were prepared by the airbrush method. The geometric electrode area was 4.0 cm^2^. Catalyst samples dispersed in ethanol were sonicated for 15 min and transferred to the vial. The density of the catalyst samples was measured using ICP-OES. 2-propanol was added to fill the total volume to 3.0 mL. The target loading density of the catalyst was 500 μg cm^−2^. Nafion ink, composed of 2-propanol (3.0 mL) and Nafion (30 μL), was airbrushed onto the catalyst. The resulting electrode was dried in a desiccator overnight.

*Electrochemical CO_2_ Reduction Reaction (eCO_2_RR) Analysis* eCO_2_RR was conducted in a handmade gas diffusion flow cell. All electrochemical data were obtained using a Biologic SP-150 potentiostat. The working electrode, reference electrode, and counter electrode were the aforementioned catalyst electrode, a Ag/AgCl (3.0 м NaCl) reference electrode, and a Pt gauze, respectively. The actual active area of the electrode was set to be 1.0 cm^2^. The cathodic and anodic chambers were separated by a Nafion 117 cation exchange membrane. A 1.0 м KOH or 1.0 м KHCO_3_ solution (50 mL) was applied to the catholyte and the anolyte separately. The CO_2_ flow rate was set to be 15 sccm for the carbon paper (CP) GDL, and 50 sccm for the PTFE GDL. The catholyte and anolyte were kept flowing at a rate of 15 mL min^−1^ for carbon paper and 18 mL min^−1^ for PTFE. Gaseous products were analyzed by gas chromatography with a pulse discharge detector (PDD) and a flame ionization detector (FID). Hydrogen, methane, and carbon monoxide were measured using PDD, and methane and ethylene were measured using FID. Liquid products were analyzed by high-performance liquid chromatography. Electrochemical potentials in the experiment were *iR*-compensated and corrected to the reversible hydrogen electrode (RHE).

$$E_{RHE}=E_{Ag/AgCl}+0.209+0.059 \times pH-iR$$

For the stability test of PTFE/Cu_2_O/Ag and CP/Cu_2_O electrodes, 250 mL of catholyte and anolyte were prepared, respectively. To maintain stable ionic environments and mitigate the cation accumulation, 200 mL of the electrolyte was periodically replaced with fresh solutions every 8 h. The gaseous products were analyzed every 20 min via gas chromatography equipped with an FID.

*In situ Potentiostatic Electrochemical Impedance Spectroscopy (PEIS) Measurement* In situ PEIS was conducted by using the identical flow cell and prepared electrodes as in the eCO_2_RR study. During the measurement, a constant potential of −0.6 V versus RHE with an alternating voltage amplitude of 10 mV was applied in 1.0 м KOH electrolyte. The impedance spectra were acquired over a frequency range of 0.1 Hz to 100 kHz, with a sampling density of 6 points per decade.

*X-ray Absorption Fine Structure (XAFS) Analysis* The XAFS experiment was conducted at Pohang Accelerator Laboratory (PAL). The beamline ring energy was 3.0 GeV, and the current was 250 mA. Two sets of Si(111) crystals were applied as monochromators. Transmittance and fluorescence measurements were used to obtain XAFS data for the reference and in situ samples, respectively. Athena software was employed for data processing and linear combination fitting (LCF) of Cu species.


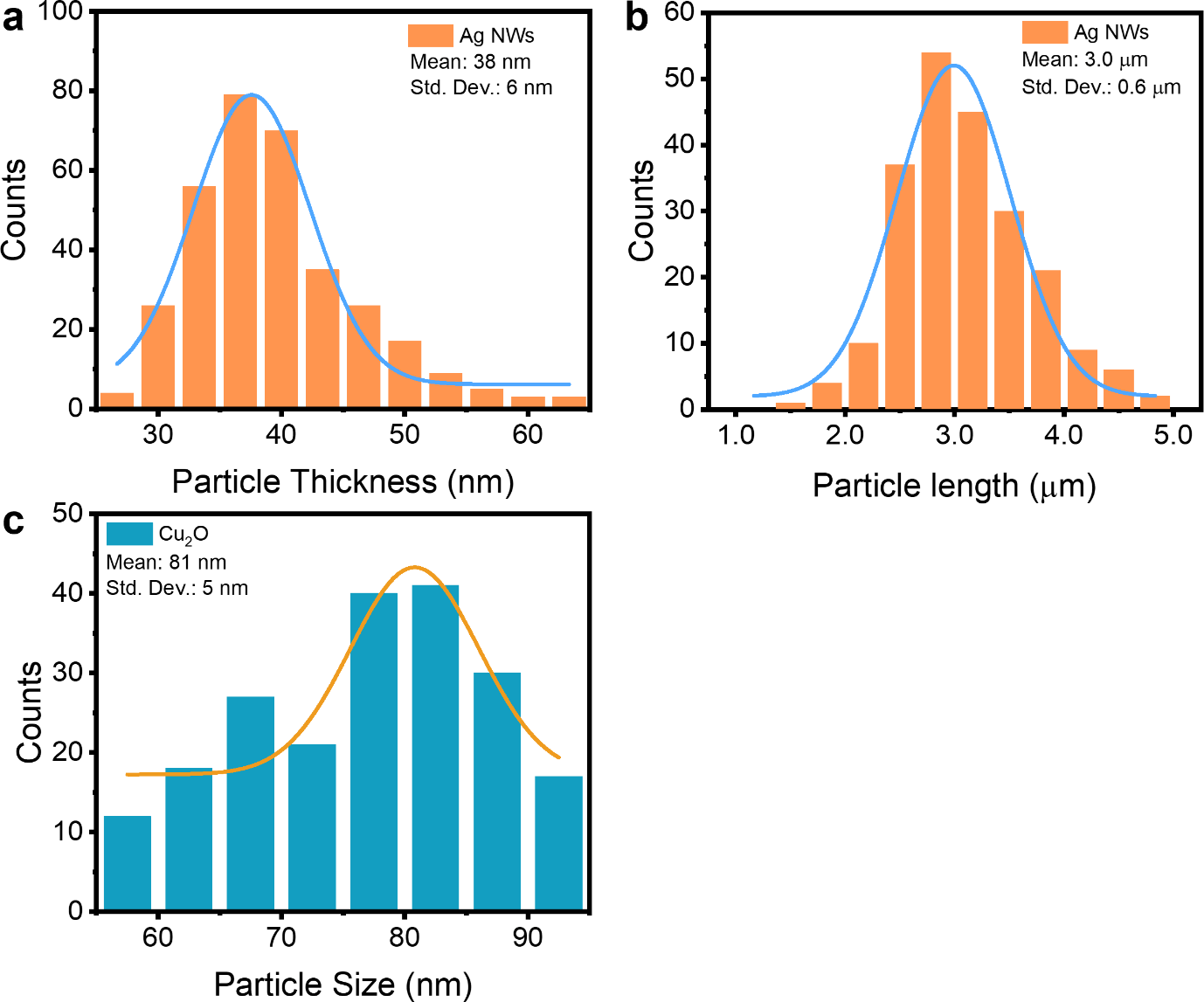


**Figure S1.** The average (a) thickness and (b) length, and their distributions of Ag NWs. (c) The average size and distribution of Cu_2_O NCs.


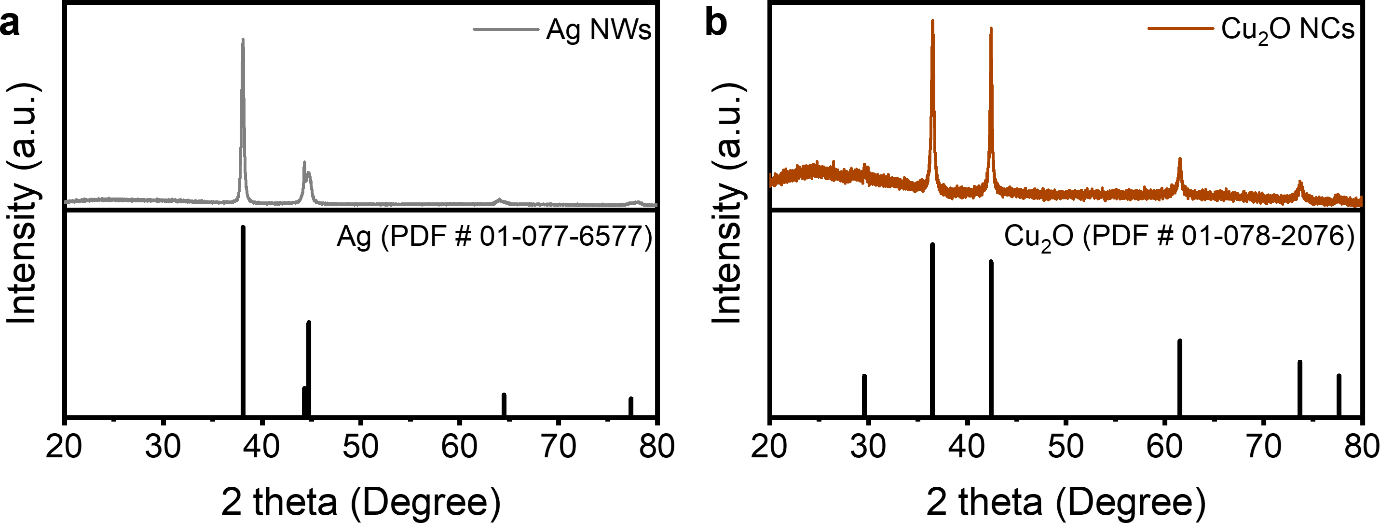


**Figure S2.** XRD patterns of (a) Ag NWs and (b) Cu_2_O NCs.

**
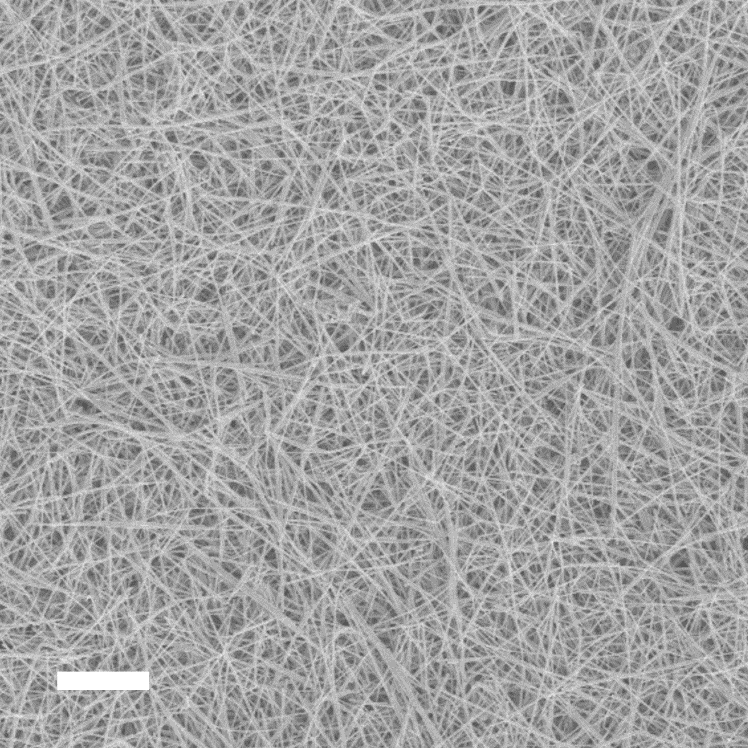
**

**Figure S3.** The top-view SEM image of the Ag NW network on PTFE/Cu_2_O/Ag. The scale bar represents 5 μm.


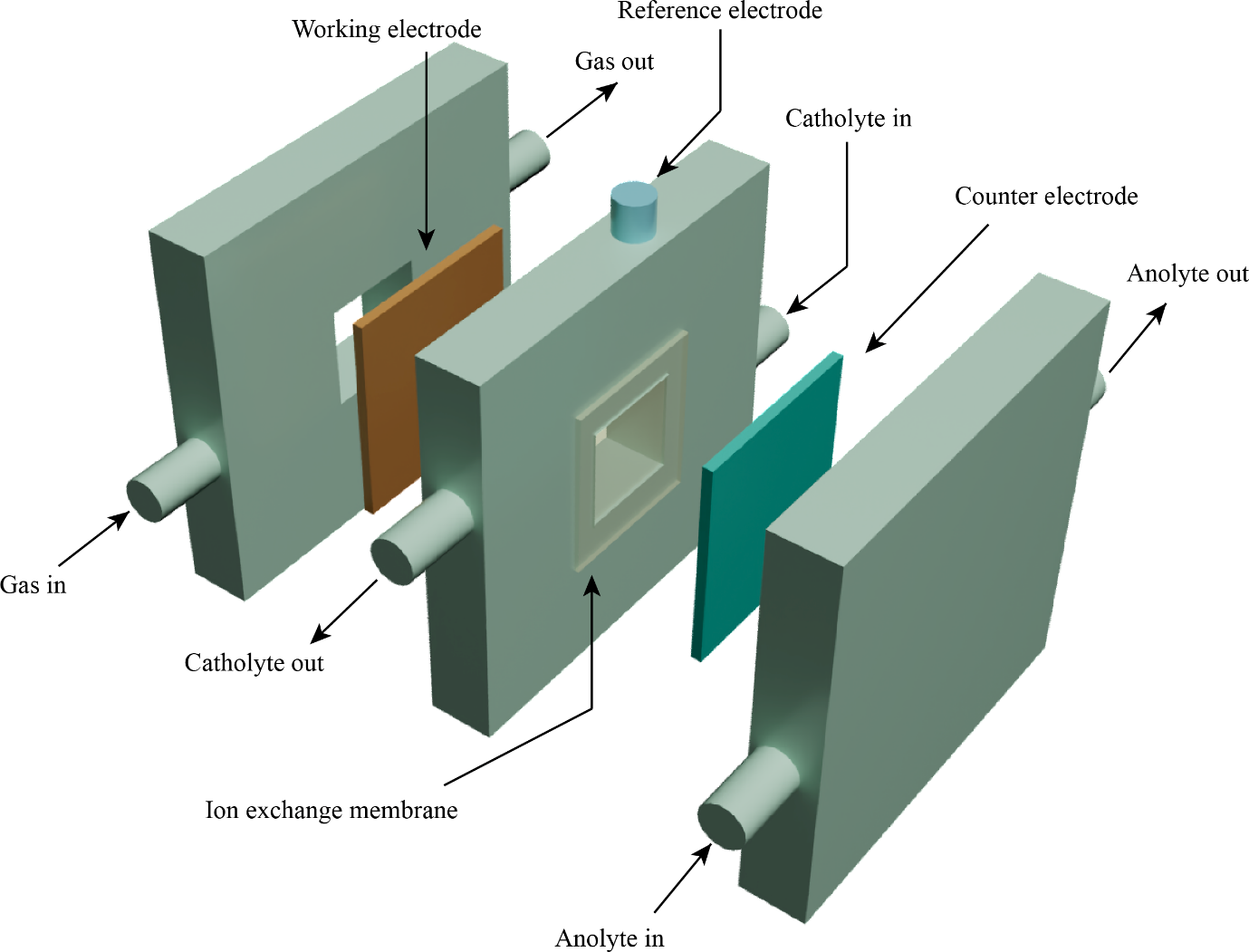


**Figure S4.** Schematic illustration of a homemade GDE flow cell.

**Table S1.** FEs and standard deviations of the products for eCO_2_RR using CP/Cu_2_O at various current densities in 1.0 м KOH.

| *j*_total_  [mA cm^–2^] | Faradaic efficiency [%] | | | | | | | |
| --- | --- | --- | --- | --- | --- | --- | --- | --- |
|  | Hydrogen | CO | Methane | Formate | Ethylene | Acetate | Ethanol | 1-Propanol |
| -400 | 21.1 ± 0.8 | 10.3 ± 1.9 | 3.8 ± 1.0 | 2.1 ± 0.1 | 38.4 ± 2.4 | 6.4 ± 1.7 | 14.9 ± 0.8 | 1.1 ± 0.3 |
| -300 | 17.0 ± 1.3 | 10.8 ± 2.0 | 3.4 ± 1.1 | 2.6 ± 0.4 | 41.5 ± 1.6 | 6.0 ± 1.4 | 14.5 ± 0.9 | 1.5 ± 0.5 |
| -200 | 17.3 ± 1.8 | 13.5 ± 2.1 | 2.7 ± 1.1 | 4.0 ± 0.1 | 43.1 ± 1.5 | 5.7 ± 1.6 | 14.0 ± 2.9 | 1.8 ± 0.3 |
| -100 | 23.3 ± 2.9 | 17.4 ± 1.4 | 1.1 ± 0.7 | 8.5 ± 0.8 | 37.4 ± 0.9 | 7.8 ± 2.1 | 8.0 ± 3.4 | 3.6 ± 1.1 |

**Table S2.** FEs and standard deviations of the products for eCO_2_RR using PTFE/Ag/Cu_2_O at various current densities in 1.0 м KOH.

| *j*_total_  [mA cm^–2^] | Faradaic efficiency [%] | | | | | | | |
| --- | --- | --- | --- | --- | --- | --- | --- | --- |
|  | Hydrogen | CO | Methane | Formate | Ethylene | Acetate | Ethanol | 1-Propanol |
| -400 | 38.8 ± 5.3 | 4.1 ± 2.2 | 8.3 ± 4.1 | 2.5 ± 1.1 | 15.0 ± 4.3 | 7.3 ± 0.7 | 14.9 ± 3.5 | 0.6 ± 0.5 |
| -300 | 33.1 ± 5.2 | 5.7 ± 2.3 | 8.0 ± 4.8 | 3.2 ± 1.5 | 20.5 ± 4.4 | 8.2 ± 1.6 | 16.9 ± 4.0 | 1.2 ± 0.2 |
| -200 | 24.1 ± 8.5 | 8.4 ± 2.2 | 4.3 ± 2.2 | 3.6 ± 1.0 | 31.7 ± 5.8 | 9.4 ± 2.1 | 22.2 ± 3.3 | 1.1 ± 0.7 |
| -100 | 14.6 ± 4.9 | 15.2 ± 3.1 | 0.5 ± 0.4 | 4.7 ± 0.5 | 41.6 ± 10.6 | 8.2 ± 3.5 | 14.9 ± 3.8 | 3.0 ± 0.8 |

**Table S3.** FEs and standard deviations of the products for eCO_2_RR using PTFE/Cu_2_O/Ag at various current densities in 1.0 м KOH.

| *j*_total_  [mA cm^–2^] | Faradaic efficiency [%] | | | | | | | |
| --- | --- | --- | --- | --- | --- | --- | --- | --- |
|  | Hydrogen | CO | Methane | Formate | Ethylene | Acetate | Ethanol | 1-Propanol |
| -400 | 15.5 ± 3.7 | 7.1 ± 1.9 | 0.5 ± 0.3 | 3.7 ± 1.8 | 43.6 ± 1.3 | 7.8 ± 0.8 | 19.8 ± 2.4 | 1.0 ± 0.1 |
| -300 | 10.4 ± 2.9 | 8.1 ± 2.0 | 0.3 ± 0.1 | 3.8 ± 1.7 | 50.5 ± 2.1 | 7.0 ± 1.0 | 19.3 ± 3.2 | 1.9 ± 0.7 |
| -200 | 7.9 ± 2.1 | 10.4 ± 2.4 | 0.2 ± 0.1 | 4.3 ± 1.5 | 53.0 ± 5.3 | 5.0 ± 0.4 | 14.9 ± 1.6 | 2.8 ± 0.3 |
| -100 | 9.8 ± 2.1 | 14.6 ± 3.7 | 0.1 ± 0.1 | 7.2 ± 1.7 | 48.1 ± 8.3 | 7.2 ± 2.8 | 9.8 ± 3.1 | 5.0 ± 1.5 |

**Table S4.** FEs and standard deviations of the products for eCO_2_RR using PTFE/Cu_2_O/CB at various current densities in 1.0 м KOH.

| *j*_total_  [mA cm^–2^] | Faradaic efficiency [%] | | | | | | | |
| --- | --- | --- | --- | --- | --- | --- | --- | --- |
|  | Hydrogen | CO | Methane | Formate | Ethylene | Acetate | Ethanol | 1-Propanol |
| -400 | 31.2 ± 4.7 | 4.3 ± 0.8 | 2.7 ± 1.6 | 1.6 ± 0.3 | 29.7 ± 1.5 | 8.4 ± 1.1 | 18.9 ± 3.8 | 1.1 ± 0.4 |
| -300 | 31.9 ± 2.0 | 3.1 ± 0.2 | 3.5 ± 2.8 | 2.2 ± 0.2 | 30.1 ± 3.4 | 8.5 ± 1.0 | 18.0 ± 2.0 | 1.3 ± 0.4 |
| -200 | 31.3 ± 4.1 | 3.3 ± 0.2 | 3.5 ± 3.1 | 3.4 ± 0.4 | 33.1 ± 6.0 | 8.5 ± 0.6 | 17.3 ± 2.7 | 1.9 ± 0.8 |
| -100 | 22.1 ± 16.7 | 5.8 ± 0.9 | 1.7 ± 1.2 | 6.8 ± 0.4 | 39.7 ± 7.2 | 9.1 ± 1.1 | 17.0 ± 3.0 | 3.1 ± 2.1 |


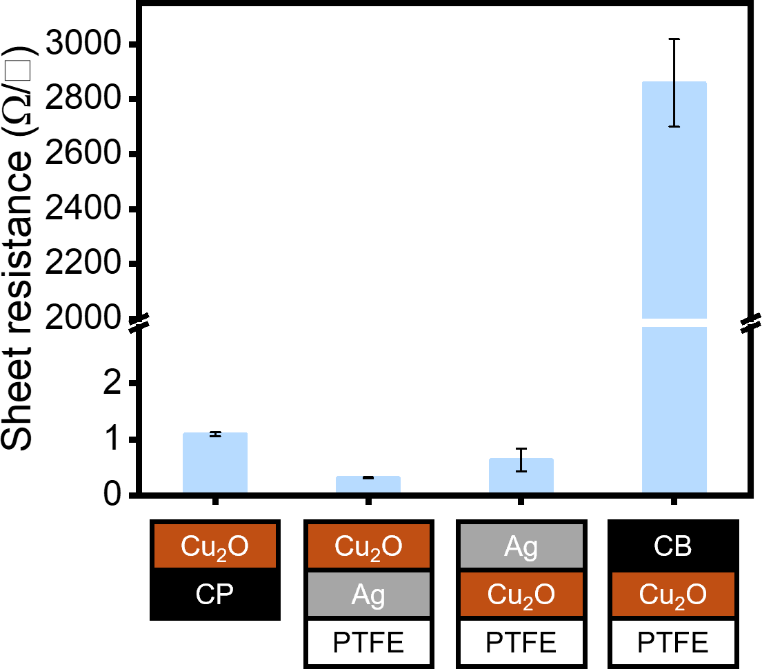


**Figure S5.** Sheet resistances of CP/Cu_2_O, PTFE/Ag/Cu_2_O, PTFE/Cu_2_O/Ag, and PTFE/Cu_2_O/CB.


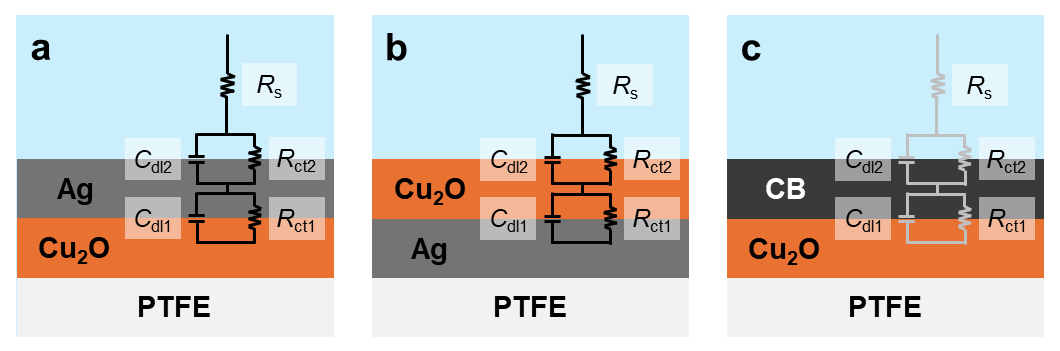


Figure S6. The equivalent circuit models of PTFE-based electrode configurations of (a) PTFE/Ag/Cu_2_O, (b) PTFE/Cu_2_O/Ag, and (c) PTFE/Cu_2_O/CB for in situ PEIS fitting. *R*_s_ denotes the Ohmic resistance. *R*_ct1_ and *R*_ct2_ represent the charge transfer resistances, and *C*_dl1_ and *C*_dl2_ represent the double layer capacitances from the high- (the buried layer) and low-frequency contributions (the overlaid layer), respectively ^[3]^.


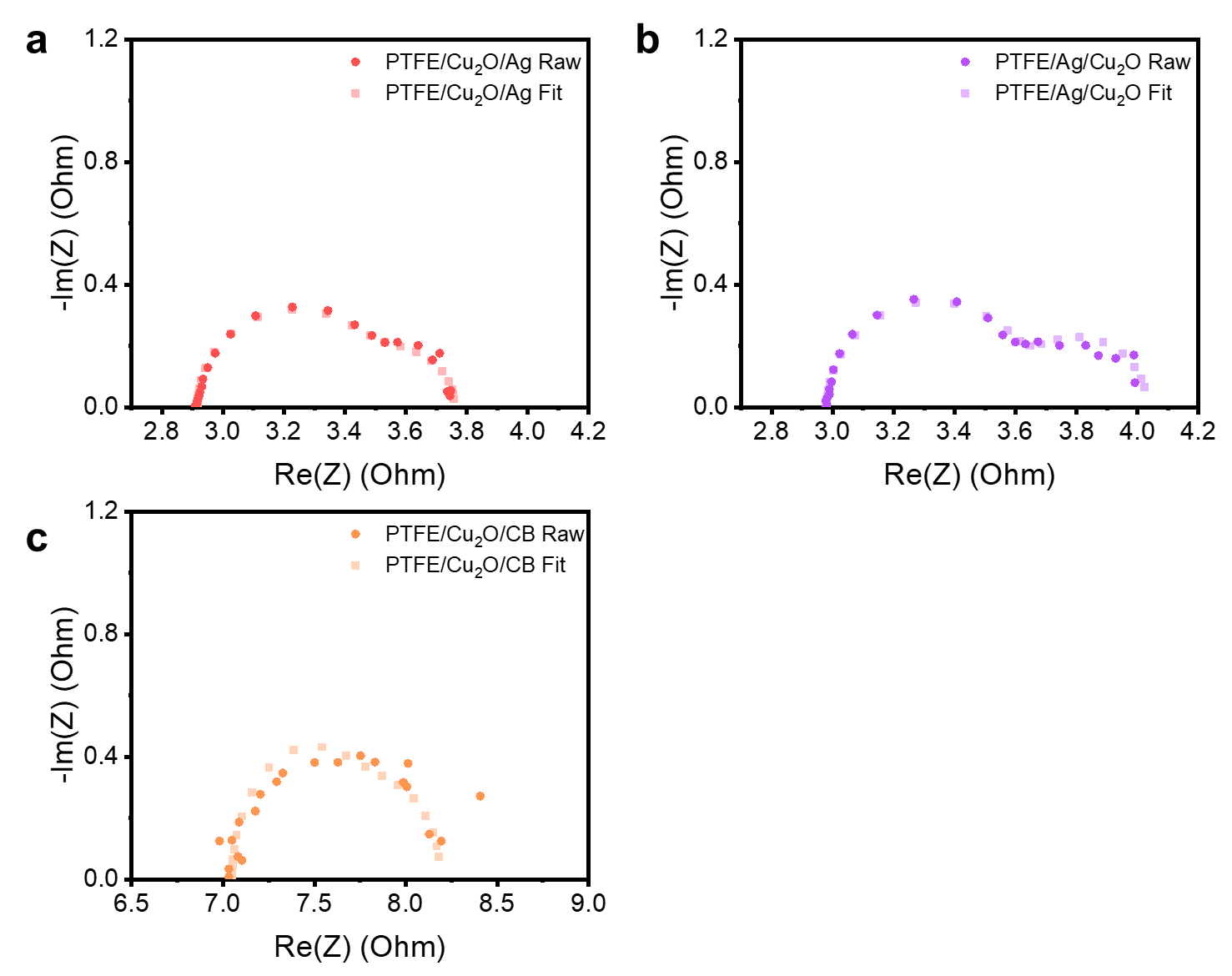


Figure S7. Nyquist plots and their fitted results of (a) PTFE/Cu_2_O/Ag, (b) PTFE/Ag/Cu_2_O, and (c) PTFE/Cu_2_O/CB at −0.6 V versus RHE in 1.0 м KOH.

**Table S5.** Fitted results of elements in the equivalent circuit model for each electrode configuration from in situ PEIS at −0.6 V versus RHE in 1.0 м KOH.

| Electrode configuration | PTFE/Cu_2_O/Ag | PTFE/Ag/Cu_2_O | PTFE/Cu_2_O/CB |
| --- | --- | --- | --- |
| *R*_s_ | 2.92 Ω | 2.98 Ω | 7.05 Ω |
| *R*_ct1_ | 0.588 Ω | 0.651 Ω | 0.751 Ω |
| *C*_dl1_ | 1.42 mF | 1.01 mF | 0.271 mF |
| *R*_ct2_ | 0.256 Ω | 0.377 Ω | 0.388 Ω |
| *C*_dl2_ | 34.0 mF | 39.0 mF | 3.70 mF |
| *χ*^2^ | 7.53 × 10^−3^ | 6.95 × 10^−3^ | 0.206 |


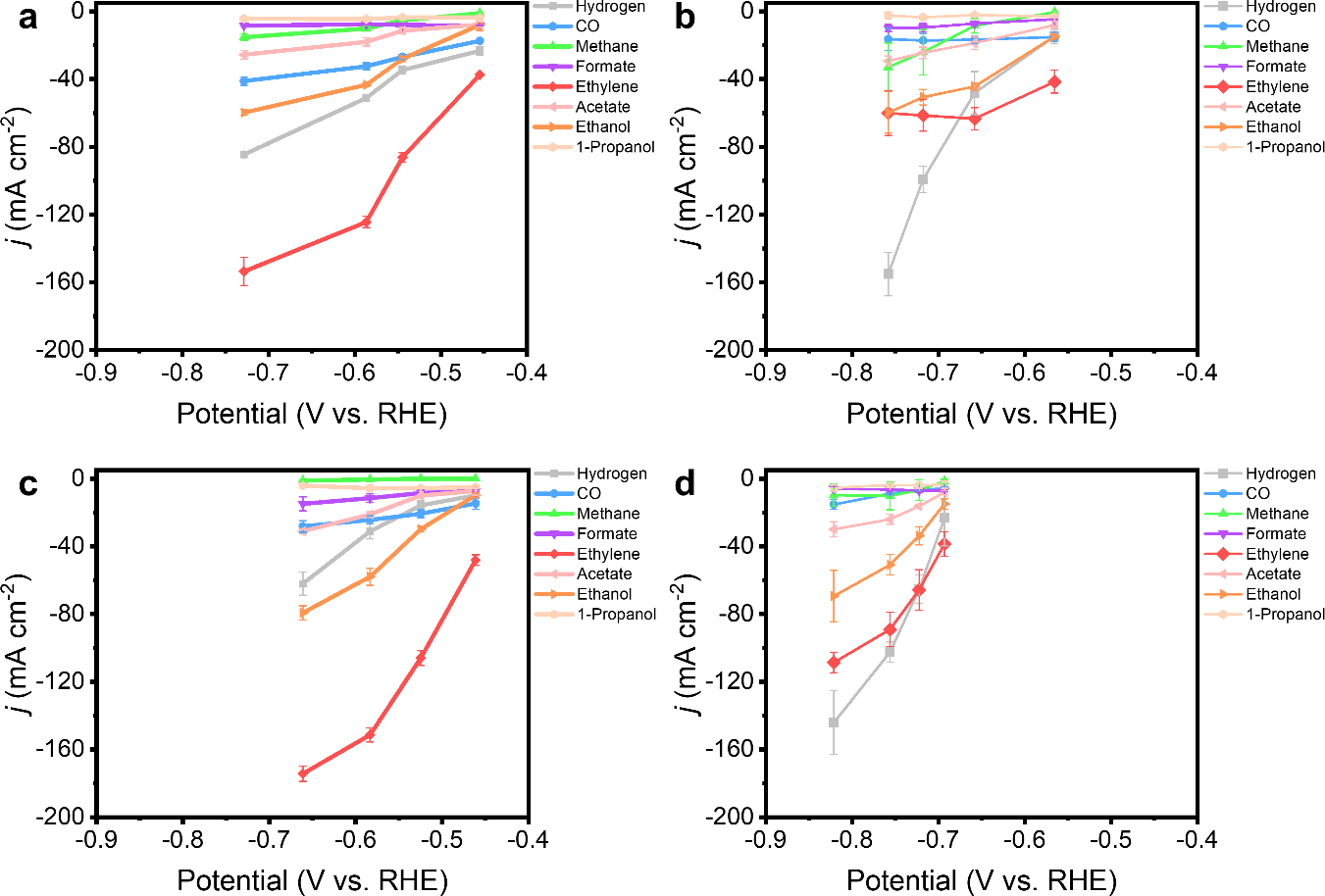


**Figure S8.** The partial current density of each product versus applied potential for (a) CP/Cu_2_O, (b) PTFE/Ag/Cu_2_O, (c) PTFE/Cu_2_O/Ag, and (d) PTFE/Cu_2_O/CB in 1.0 м KOH.

**Table S6.** Comparison of FE_C2+_ and applied potential for eCO_2_RR using PTFE/Cu_2_O/Ag under alkaline electrolytes with Cu-based catalysts reported in literature, as in **Figure 3e**.

| Catalyst | Potential  (V vs. RHE) | FE_C2+_ (%) | Electrolyte | Cell type | Reference |
| --- | --- | --- | --- | --- | --- |
| **PTFE/Cu_2_O/Ag** | **−0.58 V** | **79** | **1 м KOH** | **Flow cell** | **This work** |
| Ce(OH)*_x_*/Cu | −0.7 V | 80 | 1 м KOH | Flow cell | [4] |
| Sputtered Cu | −0.7 V | 42 | 1 м KOH | Flow cell | [5] |
| Cu-nr/CC3 | −0.9 V | 76 | 1 м KOH | Flow cell | [6] |
| Cu/PTFE | −1.85 V | 47 | 1 м KOH | Flow cell | [7] |
| Cu/NICC | −0.8 V | 77 | 1 м KOH | Flow cell | [8] |
| Cu_3_N*_x_* | −1.15 V | 70 | 1 м KOH | Flow cell | [9] |
| Cu-Al | −2 V | 88 | 1 м KOH | Flow cell | [10] |

**
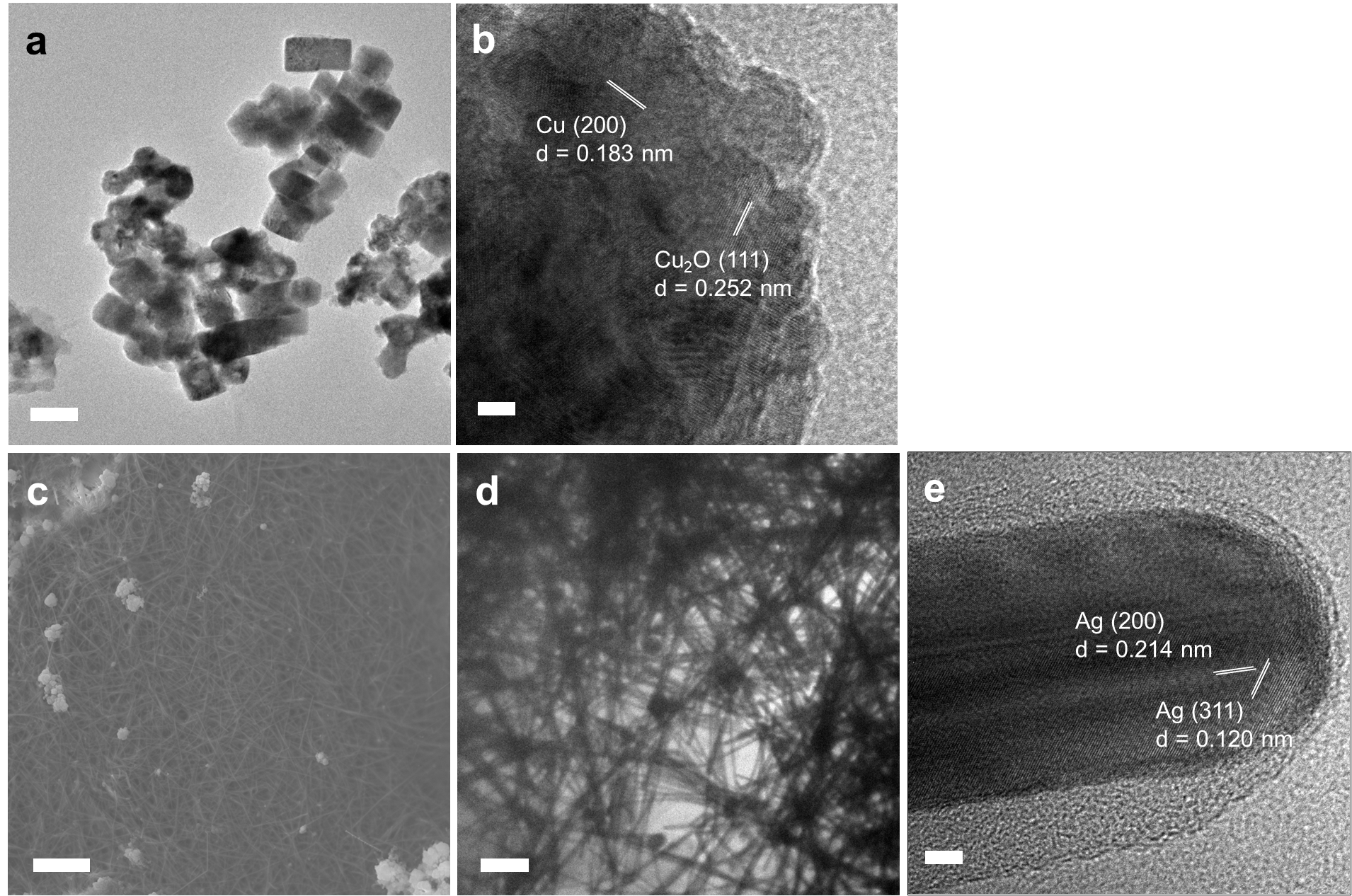
**

**Figure S9.** (a) TEM and (b) HRTEM images of Cu_2_O NCs after the 50 h stability test in 1.0 м KOH. (c) SEM, (d) TEM and (e) HRTEM images of Ag NW networks after the stability test in 1.0 м KOH. The scale bars represent (a) 100 nm, (b) 4 nm, (c) 3 μm, (d) 500 nm, and (e) 4 nm, respectively.

**Figure S10**. XRD patterns of PTFE/Cu_2_O/Ag after the 50 h stability test in 1.0 м KOH.


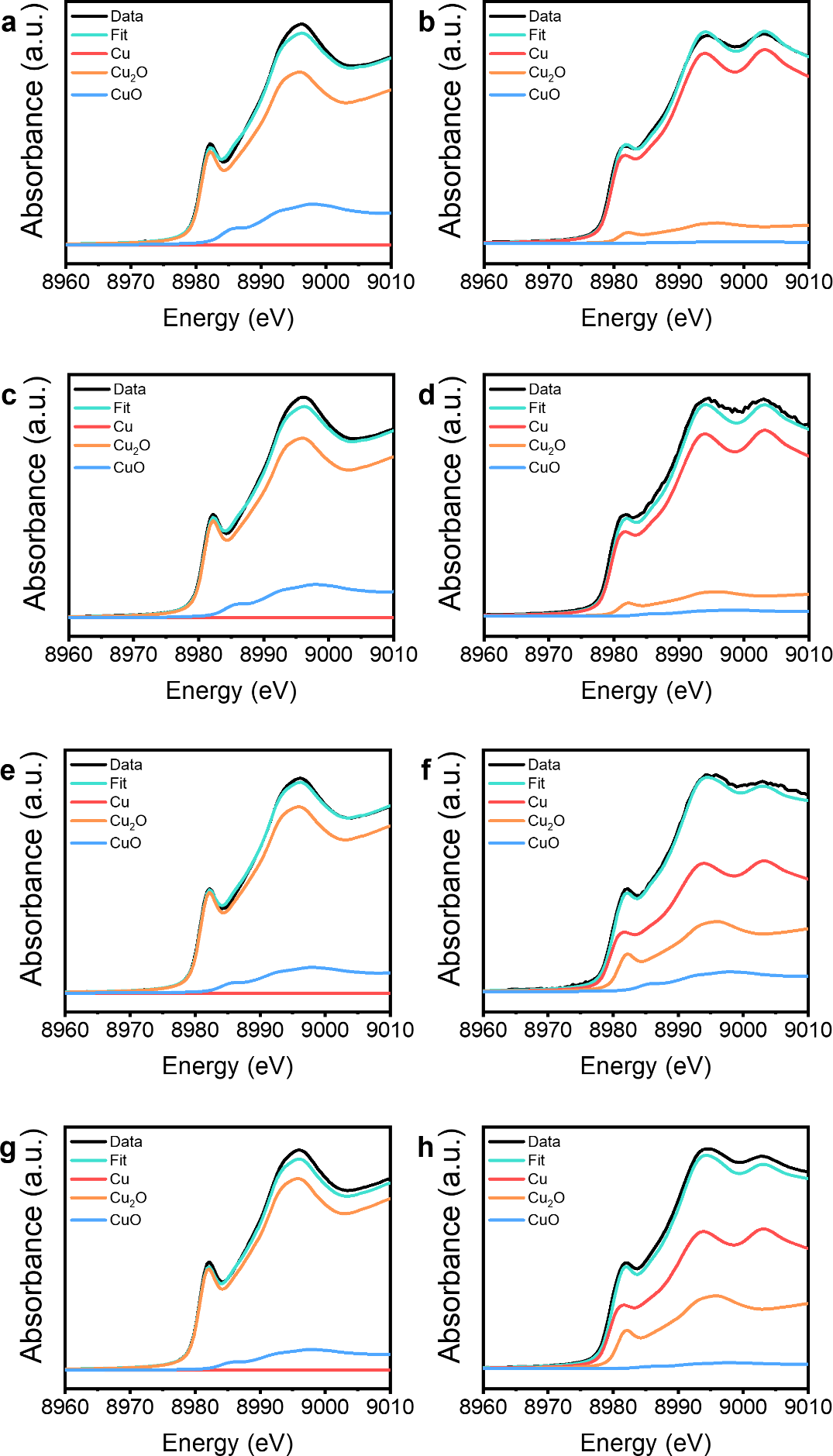


**Figure S11.** The linear combination fitting (LCF) results for Cu species of all electrodes: (a) CP/Cu_2_O as prepared, (b) CP/Cu_2_O under eCO_2_RR, (c) PTFE/Ag/Cu_2_O as prepared, (d) PTFE/Ag/Cu_2_O under eCO_2_RR, (e) PTFE/Cu_2_O/Ag as prepared, (f) PTFE/Cu_2_O/Ag under eCO_2_RR, (g) PTFE/Cu_2_O/CB as prepared, and (h) PTFE/Cu_2_O/CB under eCO_2_RR.

**Table S7.** Summary of LCF results for Cu species in all electrodes

| Sample name | | Cu (%) | Cu_2_O (%) | CuO (%) | R-factor | Reduced  chi-square |
| --- | --- | --- | --- | --- | --- | --- |
| As prepared | CP/Cu_2_O | 0 | 82.3 | 17.7 | 0.002 | 0.0003 |
|  | PTFE/Ag/Cu_2_O | 0 | 85.6 | 14.4 | 0.002 | 0.0004 |
|  | PTFE/Cu_2_O/Ag | 0 | 88.9 | 11.1 | 0.0005 | 0.00009 |
|  | PTFE/Cu_2_O/CB | 0 | 91.1 | 8.9 | 0.003 | 0.0006 |
| Under CO_2_RR | CP/Cu_2_O | 91.7 | 8.3 | 0 | 0.003 | 0.0005 |
|  | PTFE/Ag/Cu_2_O | 86.6 | 10.9 | 2.5 | 0.006 | 0.001 |
|  | PTFE/Cu_2_O/Ag | 61.0 | 31.0 | 8.0 | 0.002 | 0.0004 |
|  | PTFE/Cu_2_O/CB | 65.4 | 32.2 | 2.4 | 0.006 | 0.001 |


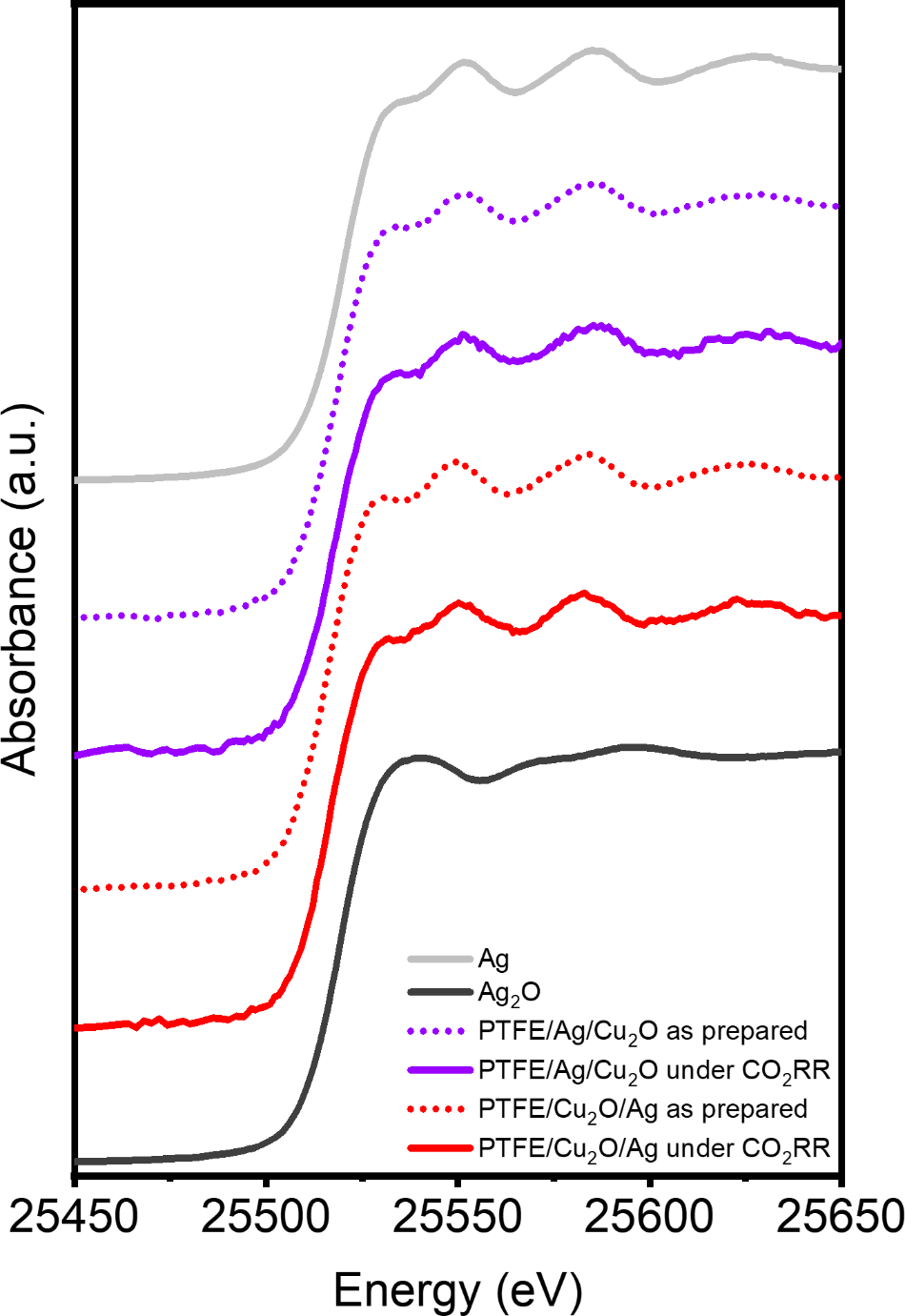


**Figure S12.** The Ag K-edge XANES for the Ag and Ag_2_O references, PTFE/Ag/Cu_2_O, and PTFE/Cu_2_O/Ag electrodes in as prepared and under eCO_2_RR conditions.


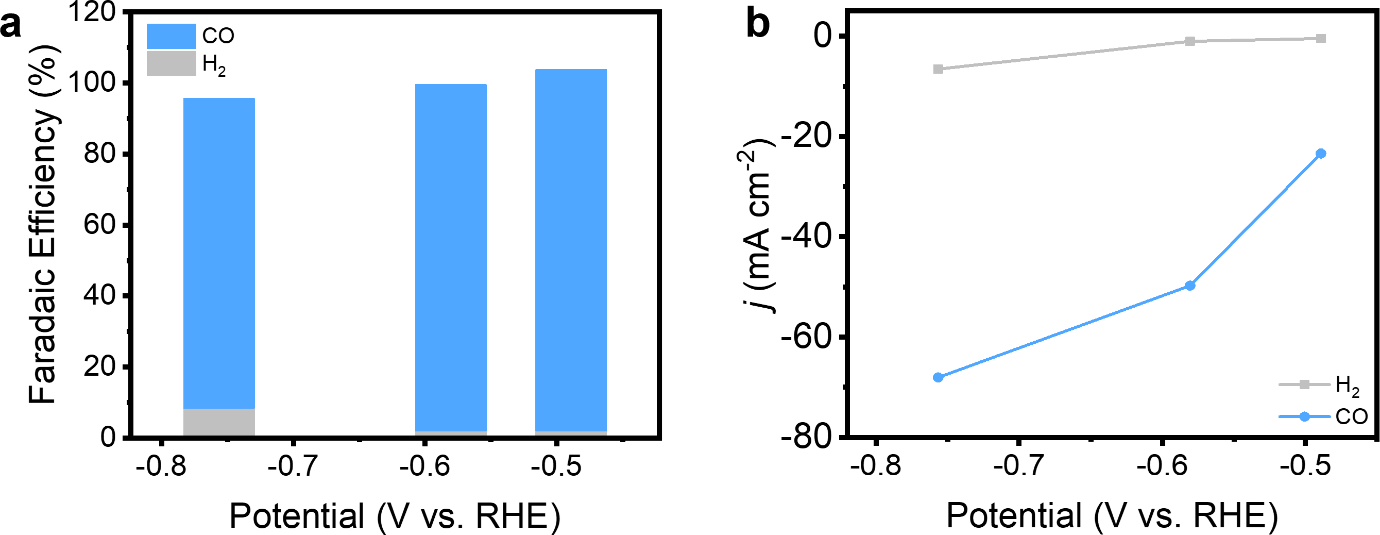


**Figure S13.** The (a) FE distribution and (b) partial current densities versus applied potential of H_2_ and CO from eCO_2_RR using PTFE/Ag electrode in 1.0 м KOH flow cell operation.

**Table S8.** FEs and standard deviations of the products for eCO_2_RR using CP/Cu_2_O at various current densities in 1.0 м KHCO_3_

| *j*_total_  [mA cm^−2^] | Faradaic efficiency [%] | | | | | | | |
| --- | --- | --- | --- | --- | --- | --- | --- | --- |
|  | Hydrogen | CO | Methane | Formate | Ethylene | Acetate | Ethanol | 1-Propanol |
| −200 | 17.8 ± 2.9 | 15.7 ± 6.7 | 2.2 ± 0.2 | 7.4 ± 3.2 | 39.3 ± 7.6 | 1.5 ± 0.7 | 13.9 ± 4.7 | 3.3 ± 0.9 |
| −150 | 19.1 ± 3.2 | 16.7 ± 7.5 | 1.6 ± 0.1 | 8.7 ± 3.0 | 39.9 ± 9.6 | 0.8 ± 0.3 | 11.6 ± 4.1 | 3.7 ± 1.1 |
| −100 | 23.4 ± 3.1 | 17.8 ± 8.0 | 1.0 ± 0.2 | 12.8 ± 2.9 | 36.5 ± 11.3 | 0.7 ± 0.4 | 9.2 ± 3.7 | 3.9 ± 2.1 |
| −50 | 33.4 ± 3.1 | 18.5 ± 6.9 | 0.2 ± 0.2 | 19.6 ± 2.6 | 27.3 ± 9.8 | 0.3 ± 0.3 | 3.3 ± 4.5 | 3.3 ± 0.6 |

**Table S9.** FEs and standard deviations of the products for eCO_2_RR using PTFE/Cu_2_O/Ag at various current densities in 1.0 м KHCO_3_

| *j*_total_  [mA cm^−2^] | Faradaic efficiency [%] | | | | | | | |
| --- | --- | --- | --- | --- | --- | --- | --- | --- |
|  | Hydrogen | CO | Methane | Formate | Ethylene | Acetate | Ethanol | 1-Propanol |
| −200 | 7.1 ± 0.8 | 4.8 ± 2.3 | 0.7 ± 1.0 | 3.5 ± 2.1 | 59.1 ± 2.7 | 3.4 ± 1.3 | 20.3 ± 1.6 | 2.9 ± 0.9 |
| −150 | 7.2 ± 1.1 | 6.4 ± 2.5 | 0.5 ± 0.6 | 4.3 ± 1.3 | 63.5 ± 3.3 | 2.3 ± 0.7 | 16.9 ± 4.1 | 4.0 ± 0.9 |
| −100 | 8.9 ± 1.5 | 9.1 ± 2.7 | 0.3 ± 0.3 | 7.0 ± 0.5 | 63.2 ± 3.2 | 1.9 ± 0.8 | 14.3 ± 3.6 | 4.1 ± 1.3 |
| −50 | 16.0 ± 2.1 | 16.2 ± 2.8 | 0.1 ± 0.2 | 11.1 ± 0.8 | 52.5 ± 4.1 | 1.1 ± 0.6 | 3.2 ± 2.2 | 4.7 ± 2.4 |


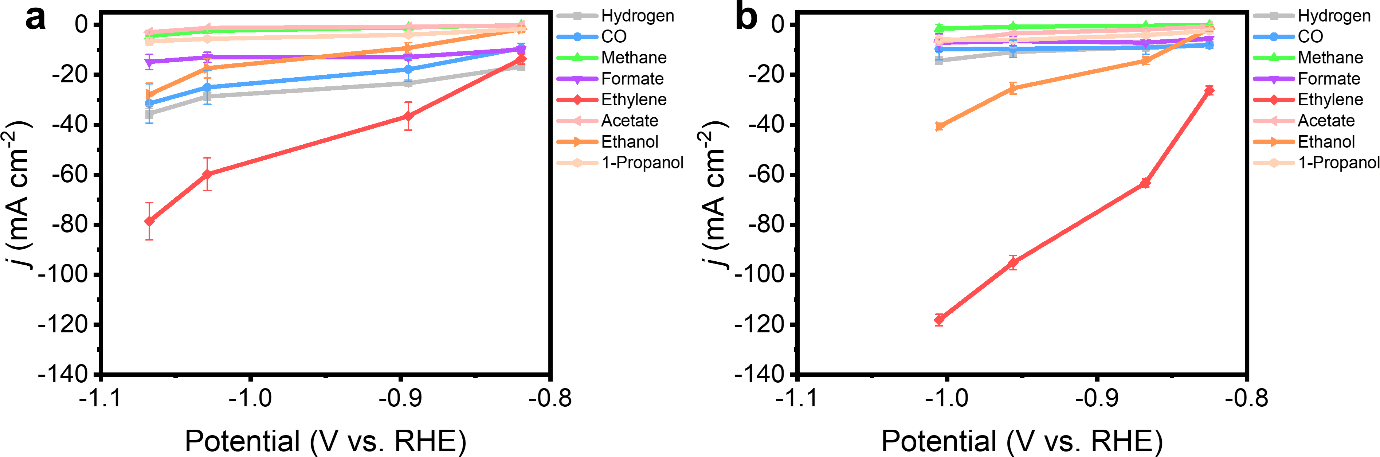


**Figure S14.** The partial current density versus applied potential of each product for (a) CP/Cu_2_O and (b) PTFE/Cu_2_O/Ag in 1.0 м KHCO_3_.


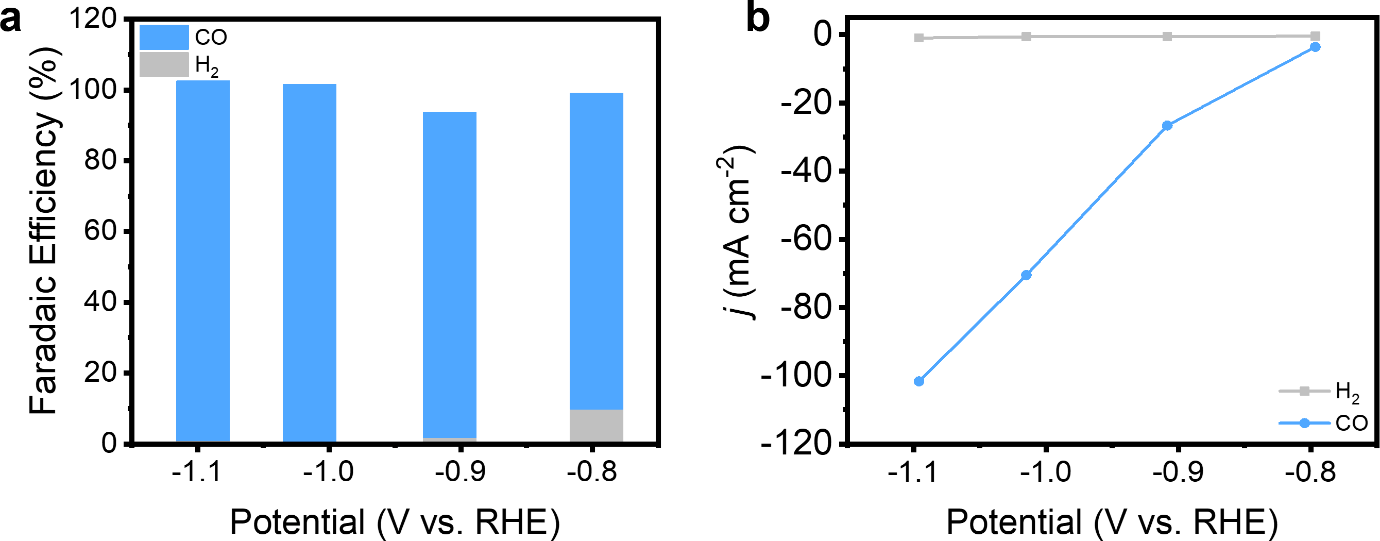


**Figure S15.** The (a) FE distribution and (b) partial current densities versus applied potential of H_2_ and CO from eCO_2_RR using PTFE/Ag electrode in 1.0 м KHCO_3_ flow cell operation.

**Table S10.** Comparison of FE_C2+_ and applied potential of the products for eCO_2_RR using PTFE/Cu_2_O/Ag under neutral electrolytes with Cu-based catalysts reported in literature, as in **Figure 6f**.

| Catalyst | Potential  (V vs. RHE) | FE_C2+_ (%) | Electrolyte | Cell type | Reference |
| --- | --- | --- | --- | --- | --- |
| **PTFE/Cu_2_O/Ag** | **−0.96 V** | **87** | **1 м KHCO_3_** | **Flow cell** | **This work** |
| Cu/GDL | −1.12 V | 80 | 2 м KCl | Flow cell | [11] |
| Cu_2_O | −1.2 V | 72 | 0.98 м KHCO_3_  + 0.02 м KI | Flow cell | [12] |
| Cu NWs/NC@Ag | −1.2 V | 82 | 2 м KCl | Flow cell | [13] |
| DMAN-Cu | −1.0 V | 80 | 1 м KHCO_3_ | Flow cell | [14] |
| U-Cu_2_O | −1.0 V | 59 | 1 м KHCO_3_ | Flow cell | [15] |
| Cu HoMSs | −0.88 V | 77 | 0.5 м KHCO_3_ | Flow cell | [16] |
| Ag/Cu_2_O/Al | −1.2 V | 81 | 1 м KHCO_3_ | Flow cell | [17] |


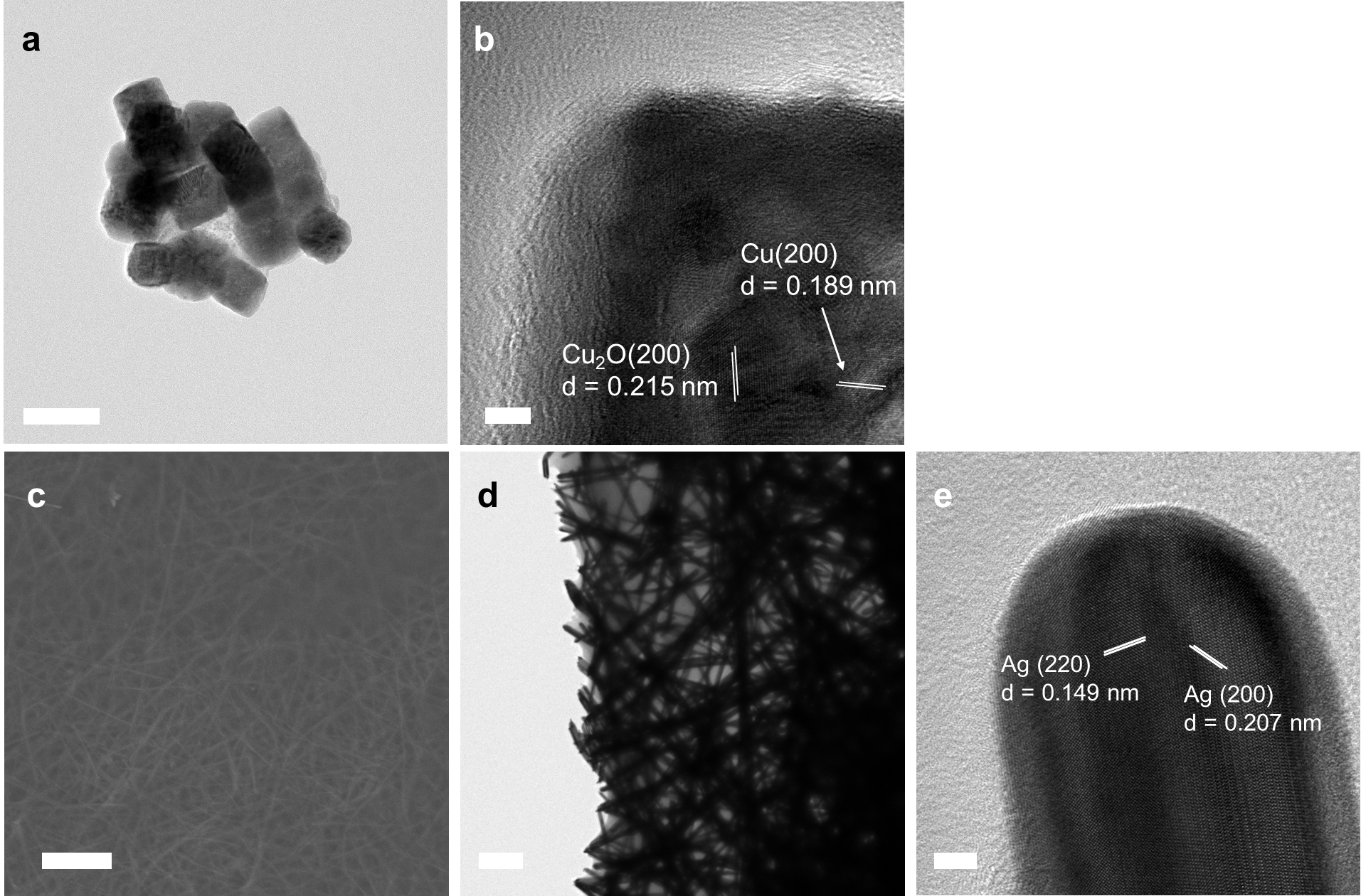


**Figure S16.** (a) TEM and (b) HRTEM images of Cu_2_O NCs after the 50 h stability test in 1.0 м KHCO_3_. (c) SEM, (d) TEM and (e) HRTEM images of Ag NWs after the stability test in 1.0 м KHCO_3_. The scale bars represent (a) 100 nm, (b) 4 nm, (c) 2 μm, (d) 400 nm, and (e) 4 nm, respectively.

**Figure S17.** XRD patterns of PTFE/Cu_2_O/Ag after the 50 h stability test in 1.0 м KHCO_3._

**References**

[1] D. Raciti, T. Braun, B. M. Tackett, H. Xu, M. Cruz, B. J. Wiley, T. P. Moffat, *ACS Catal.* **2021**, *11*, 11945.

[2] J. Kim, W. Choi, J. W. Park, C. Kim, M. Kim, H. Song, *J. Am. Chem. Soc.* **2019**, *141*, 6986.

[3] J. Zeng, K. Bejtka, W. Ju, M. Castellino, A. Chiodoni, A. Sacco, M. A. Farkhondehfal, S. Hernández, D. Rentsch, C. Battaglia, C. F. Pirri, *Appl. Catal. B Environ.* **2018,** *236,* 475.

[4] M. Luo, Z. Wang, Y. C. Li, J. Li, F. Li, Y. Lum, D.-H. Nam, B. Chen, J. Wicks, A. Xu, T. Zhuang, W. R. Leow, X. Wang, C.-T. Dinh, Y. Wang, Y. Wang, D. Sinton, E. H. Sargent, *Nat. Commun.* **2019**, *10*, 5814.

[5] F. Huq, I. Sanjuán, S. Baha, M. Braun, A. Kostka, V. Chanda, J. R. C. Junqueira, N. Sikdar, A. Ludwig, C. Andronescu, *ChemElectroChem* **2022**, *9*, e202101279.

[6] C. Chen, X. Yan, Y. Wu, S. Liu, X. Zhang, X. Sun, Q. Zhu, H. Wu, B. Han, *Angew. Chem. Int. Ed.* **2022**, *61*, e202202607.

[7] K. J. Puring, D. Siegmund, J. Timm, F. Möllenbruck, S. Schemme, R. Marschall, U.-P. Apfel, *Adv. Sustain. Syst.* **2021**, *5*, 2000088.

[8] H.-P. Iglesias van Montfort, M. Li, E. Irtem, M. Abdinejad, Y. Wu, S. K. Pal, M. Sassenburg, D. Ripepi, S. Subramanian, J. Biemolt, T. E. Rufford, T. Burdyny, *Nat. Commun.* **2023**, *14*, 6579.

[9] C. Peng, G. Luo, Z. Xu, S. Yan, J. Zhang, M. Chen, L. Qian, W. Wei, Q. Han, G. Zheng, *Adv. Mater.* **2021**, *33*, 2103150.

[10] M. Zhong, K. Tran, Y. Min, C. Wang, Z. Wang, C.-T. Dinh, P. De Luna, Z. Yu, A. S. Rasouli, P. Brodersen, S. Sun, O. Voznyy, C.-S. Tan, M. Askerka, F. Che, M. Liu, A. Seifitokaldani, Y. Pang, S.-C. Lo, A. Ip, Z. Ulissi, E. H. Sargent, *Nature* **2020**, *581*, 178.

[11] X. Zhang, J. Li, Y.-Y. Li, Y. Jung, Y. Kuang, G. Zhu, Y. Liang, H. Dai, *J. Am. Chem. Soc.* **2021**, *143*, 3245.

[12] X. Lv, Y. Yang, J. Lv, L. Ji, J. Wang, X. Wu, Z. Li, X. Li, Q. Liu, Z. Qi, Q. Lin, A. Wu, H. B. Wu, *Adv. Func. Mater.* **2024**, *34*, 2311236.

[13] L. Bian, Y. Bai, J.-Y. Chen, H.-K. Guo, S. Liu, H. Tian, N. Tian, Z.-L. Wang, *ACS Nano* **2025**, *19*, 9304.

[14] L. Fan, C.-Y. Liu, P. Zhu, C. Xia, X. Zhang, Z.-Y. Wu, Y. Lu, T. P. Senftle, H. Wang, *Joule* **2022**, *6*, 205.

[15] T. Möller, F. Scholten, T. N. Thanh, I. Sinev, J. Timoshenko, X. Wang, Z. Jovanov, M. Gliech, B. R. Cuenya, A. S. Varela, P. Strasser, *Angew. Chem. Int. Ed.* **2020**, *59*, 17974.

[16] C. Liu, M. Zhang, J. Li, W. Xue, T. Zheng, C. Xia, J. Zeng, *Angew. Chem. Int. Ed.* **2022**, *61*, e202113498.

[17] S. Yamaguchi, H. Ebe, T. Minegishi, M. Sugiyama, *ACS Appl. Mater. & Interfaces* **2024**, *16*, 17371.
